# When Reality Defies Prediction: Polymorphism, Twinning, and Accordion Crystals

**DOI:** 10.1021/jacs.5c22213

**Published:** 2026-01-27

**Authors:** Amy V. Hall, Alice C. Taylor, Natalie E. Pridmore, Aurora J. Cruz-Cabeza, David K. Smith, Niccolò Cosottini, Mark A. Fox, Amrita Chattopadhyay, Stefanos Konstantinopoulos, Daniel N. Rainer, Simon J. Coles, Nicholas Blagden, Qi Zhang, Leon Bowen, Toby J. Blundell

**Affiliations:** † Department of Chemistry, 3057 Durham University, Stockton Road, Durham DH1 3LE, U.K.; ‡ Department of Chemistry, 8748 University of York, Heslington, York YO10 5DD, U.K.; § School of Chemistry and Chemical Engineering, 7423 University of Southampton, Highfield, Southampton SO17 1BJ, U.K.; ∥ State Key Laboratory of Chemical Engineering, 47860 East China University of Science and Technology, Shanghai 200237, China; ⊥ Department of Physics, Durham University, South Road, Durham DH1 3LE, U.K.

## Abstract

The ability to understand crystallization and predict the resulting solid form of a system is not always easily achieved, but it is critical, particularly in the field of materials science. Intriguing (and previously unreported) crystallization behavior is observed with terephthalic dihydrazide (TeDi) as it rapidly forms two concomitant crystalline polymorphs upon cooling in solution. The crystal morphology of Form I (FI) has not been seen before in organic systems and involves impressive, accordion-like stacks, composed of numerous twin domains and remains stable in solution for years. Form II (FII) exists as large needles that disappear in solution after 20 h. All experimental methods employed reveal that FI is the most stable polymorph. Conversely, all computational methods utilized (conformational analyses, lattice energy calculations, and crystal structure prediction) suggest that FII is the most stable polymorph. Isolation of FII was achieved by the crystallization of TeDi powder with a supramolecular mimetic gelator, as the gel fibers act as a template for the preferential crystallization of FII, due to the comparable crystal packing of FII and the gelator. This work highlights the impact of crystallization behavior in a real laboratory and the defects, disorder, and twinning that lead to remarkable crystal morphologies that may not be accounted for with idealized calculations, and also explores approaches for controlling and directing crystallization outcomes.

## Graphical abstract

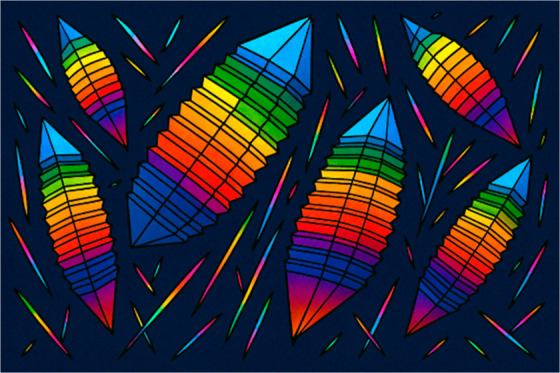

## Introduction

The nucleation and growth of crystals in solution are critical steps in the crystallization pathway that can control the resulting crystal quantity, size, morphology, and polymorphism of a system.^1–3^ Polymorphs, defined as different arrangements of a compound in the solid state, can be notoriously difficult to control. In the pharmaceutical industry, loss of polymorphic control can have detrimental impacts, with ritonavir as the most infamous example.^4^ Attempts to understand polymorph stability and exert polymorph control have been made through various experimental routes in solution. Examples include temperature effects, solvent choice, pH change, and the use of additives (all examples are summarized in a recent review)^5^ but also through less conventional routes, such as mechanochemical milling,^6^ ultrasound application,^7,8^ and supramolecular gelation.^9,10^ Supramolecular gels that assemble from low-molecular-weight gelators (LMWGs) as a result of non-covalent interactions^11^ have been proven as a versatile tool to search for new and metastable polymorphs.^12^ Supramolecular gelators can be specifically designed to structurally mimic a target compound, with pharmaceuticals as a common example.^13,14^ Supramolecular mimetic gelators have also been used to separate concomitant drug polymorphs, and it has been argued this is due to preferential nucleation sites on the self-assembled gel fibers.^15,16^

A group of compounds with multiple hydrogen bond donor and acceptor functionalities that have long been neglected for their polymorphism potential are simple *bis*(acylhydrazides), with only the simplest (oxalyl dihydrazide, Figure 1) known to exhibit polymorphism. Oxalyl dihydrazide forms five polymorphs (Cambridge Structural Database (CSD)^17^ refcodes VIPKIO01-05) due to variation of the torsion angle around the NH–NH_2_ bond.^18,19^ Most impressively, each of the five polymorphs undergo a further phase change under high-pressure conditions.^20^ This rich polymorphic behavior suggests that other simple *bis*(acylhydrazides) may behave similarly, shedding light on the origins of polymorphism in hydrogen-bonding dominated systems. Furthermore, acylhydrazides play important roles in pharmaceutical and agrochemical industries^21,22^ and are also increasingly important components as reactive and dynamic building blocks in synthetic and supramolecular chemistry.^23^ As such, understanding, controlling, and manipulating their structural behavior are of great significance in materials science. A marker of the nascent stage of structural research on *bis*(acylhydrazides) is the fact that a very simple *bis*(acylhydrazide) with a *para*-substituted phenylene linker, terephthalic dihydrazide (TeDi, Figure 1), has not yet been structurally characterized. Based on the plethora of oxalyl dihydrazide polymorphs, we anticipate TeDi to exhibit rich solid-state behavior. In the present work, we focus on understanding TeDi using a multidisciplinary approach, combining comprehensive experimental characterization with extensive computational techniques. We realize that TeDi is indeed polymorphic, though there is an unexpected tension between the experimentally observed relative stability of its polymorphs and high-level outcomes obtained using cutting-edge DFT-d methods, thus highlighting the need for continued development of theoretical tools for polymorph analysis. TeDi Form I also exhibits a remarkable accordion-like morphology of a type only known to inorganic systems, such as potash alum^24^ and quartz,^25,26^ but it is unprecedented in organic crystals. Finally, we show that the polymorphic behavior of TeDi can be controlled by crystallization from a supramolecular gel based on a substrate-mimetic *bis*(acylhydrazide) gelator.

**Figure 1:**
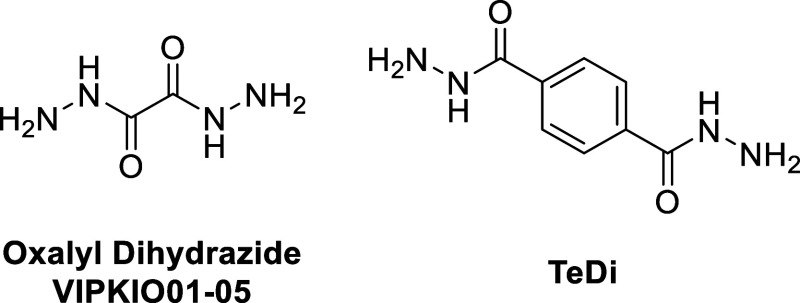
The chemical structure of oxalyl dihydrazide with CSD refcodes VIPKIO01-05 ^18^ (left) and terephthalic dihydrazide, TeDi (right).

## Results and Discussion

### Crystal Growth and Polymorphism

The concomitant crystallization of different crystal morphologies occurs when a supersaturated solution of TeDi at 100 °C in water is allowed to cool to room temperature in a sealed vial. The fast nucleation and crystal growth of TeDi result in the formation of two final morphologies: Form I (FI) and Form II (FII). FI begins as seemingly single, thin plates and rapidly develops into blocks, then as visibly layered, accordion crystals of different sizes within 10 min of cooling (Figure 2). FII begins as small needles, which quickly grow significantly larger than the FI accordion crystals, but the FII needles disappear after standing for 20 h in solution, whereas the FI accordions remain unchanged in solution for years. The accordion and needle morphologies reproducibly form with each cooling crystallization and are consistent across low- and high-supersaturation ranges.

**Figure 2:**
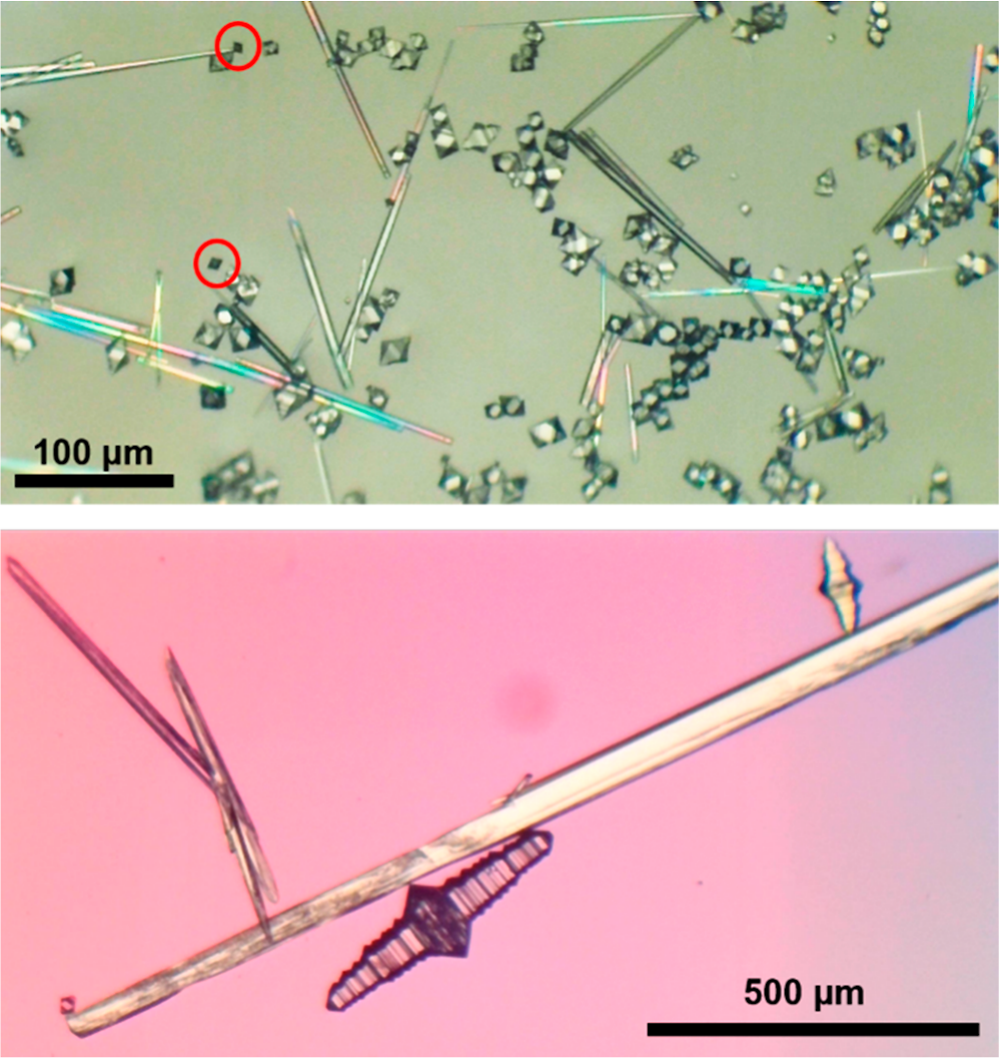
Top: Optical microscopy images of TeDi Top: small FI plates (circled), FI blocks of different sizes, small FI accordion crystals, and FII needles, obtained by fast cooling a supersaturated solution of TeDi in water. Bottom: the final morphologies of FI (accordion) and FII (large needles) crystals in water.

Single-crystal X-ray diffraction of the accordions and needles reveals that the different morphologies correspond to two different packing polymorphs of TeDi. The crystallographic information for FI and FII is given in Table 1. The hydrogen bonding and crystal packing environments of TeDi are dramatically different in the two polymorphs (Figure 3). Descriptions of the molecular arrangements and intermolecular interactions of FI and FII can be found in the Supporting Information, along with hydrogen bond distances and their estimated standard deviations (Table S1).

**Table 1:** Crystallographic Details of the TeDi Polymorphs

polymorph	form I	form II
formula	C_8_H_10_N_4_O_2_	C_8_H_10_N_4_O_2_
formula weight (g/mol)	194.20	194.20
morphology	accordion	needles
crystal color	colorless	colorless
temperature (K)	120.0(2)	120.0(2)
crystal system	monoclinic	monoclinic
space group	*P*2_1_/*c*	*P*2_1_/*n*
space group	*P*2_1_/*c*	*P*2_1_/*n*
*a* (Å)	8.0647(15)	6.1936(16)
*b* (Å)	13.169(2)	3.7771(10)
*c* (Å)	8.1042(15)	18.025(5)
β (°)	104.289(6)	94.485(9)
volume (Å^3^)	834.1(3)	420.39(19)
*Z*	4	2
final *R*_1_ indices [I ≥ 2σ (I)]	0.0656	0.0659
final GooF	1.095	1.0618

**Figure 3:**
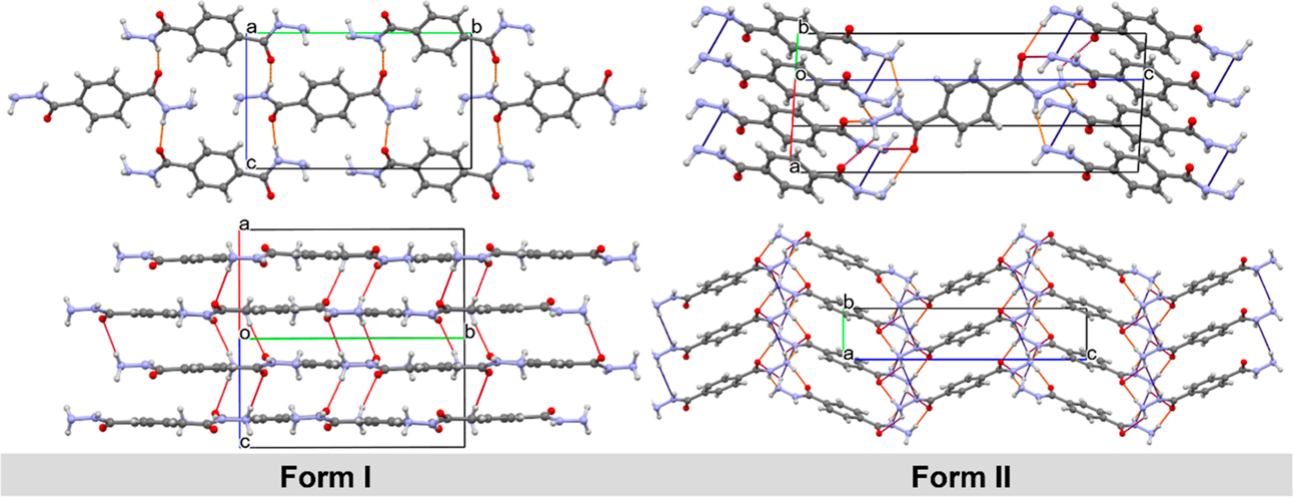
The X-ray structures of FI (left) and FII (right) and their hydrogen-bonded environments and crystal packing. Hydrogen bonds are colored by distance: short (yellow), mid (red), and long (blue).

X-ray diffraction also reveals that FI contains pseudo-merohedral twinning, with a twin law of (0 0 1, 0–1 0, 1 0 0), corresponding to a 180° rotation around the *b*-axis. The parallel arrangement of the twin domains in this manner creates a series of very thin, repeated alternating layers, such as those in potash alum,^24^ and in polysynthetic twinned crystals (observed in naturally occurring feldspar minerals). The presence of multiple alternating twin lamellae is related by one twin law in the accordion crystals. Polysynthetic twinning is well-known in inorganic minerals and, to the best of our knowledge, has not been observed before in molecular organic crystals. One would expect the diffraction pattern of the FI accordions to be polycrystalline due to the multiple lamellae that they possess; however, the reciprocal lattice slices displayed in Figure 4 reveal that this is not the case.

**Figure 4:**
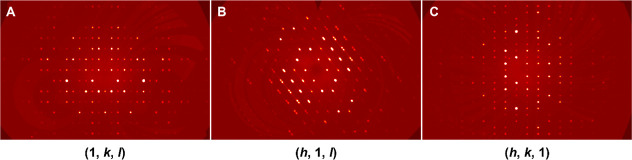
The reflections of an FI accordion crystal in reciprocal space along different planes.

Eight of the small FI plate crystals were analyzed at the Diamond Light Source, and only one crystal exhibited no observable twinning; the thin FI plates are also very unstable in solution, suggesting that the non-twinned FI plates are metastable in comparison to the FI accordions. Structures with twinning are modeled as the major component only, defined by the twin law (i.e., not physically modeled), thus the non-twinned FI structure otherwise appears identical to the other twinned FI structures (see Supporting Information for the crystallographic details for the non-twinned FI plates, twinned FI blocks, and twinned FI accordions). Twin scale factors were determined for the crystals analyzed, with blocks displaying a twin ratio close to equal at 49:51, while the accordions have a twin ratio close to 55:45 for each domain present in the crystal. A twin scale factor was not determined for the twinned plate crystals due to poor data quality; however, indexing these crystals and the accordion crystals reveals that the largest face of the crystal, corresponding to the (1̅01) plane, is also the crystal stacking direction, likely in order to minimize the energy of this face (Figure 5). The role of stacking faults within the lamellar FI crystals has also been considered, with the possibility of twinning and stacking faults occurring together, as with (±)-modafinil.^27^ The presence of stacking faults has an associated energetic penalty (stacking fault energy); however, stacking faults can be difficult to characterize and have rarely been observed in organic materials.

**Figure 5:**
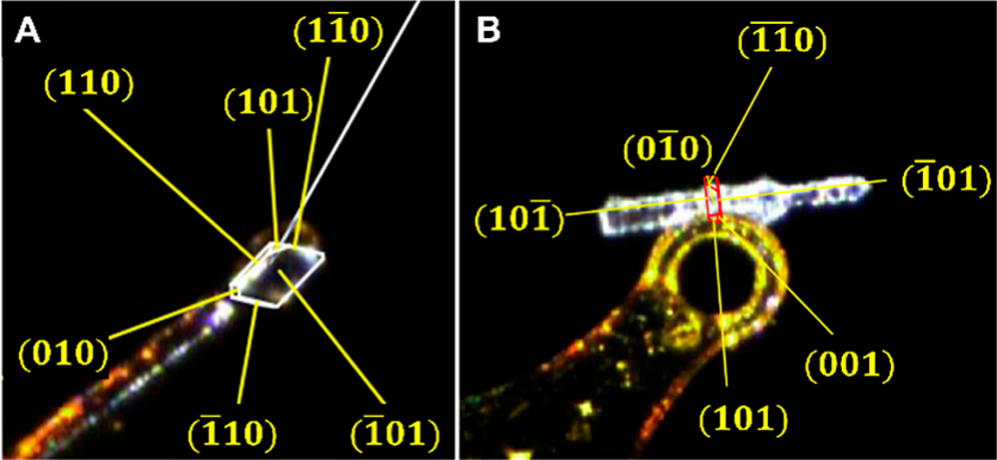
Indexing of the FI (A) plate and (B) accordion crystals, revealing the (1̅01) plane and the large face of the plate crystal is the growth direction.

Scanning electron microscopy (SEM) images of the FI accordion crystals detail the numerous and very thin layers that the accordions are composed of (Figure 6A,B), with the same layered structures also apparent in the commercially available crystalline powder of TeDi (Figure 6C,D) which has a powder X-ray diffraction (PXRD) pattern matching that of the FI accordions (Figure S1). A related compound to TeDi, terephthalic acid, also exhibits a twinned structure that displays polymorphism (though with thermosalient properties);^28–30^ however, the striated morphology of the terephthalic acid crystals is not comparable to the large corrugated stacks of FI TeDi.^31,32^ An accordion-like crystal morphology has been observed in thin perovskites,^33^ while the Tessin and Muzo habits of quartz show an analogous lamellar crystal morphology to FI accordions, whereby the crystal layers become continuously thinner toward the tip of the crystal.^25,26^ In the case of TeDi FI accordions, each of the multilayer stacks are non-uniform in width and length, another feature of these crystals that is uncommon, particularly in organic systems. In addition to TeDi FI accordion crystals, unusual morphologies have been observed with curved benzil crystals,^34^ and in inorganic systems, complex morphologies in molluscan shell mineralization,^35^ and even star-^36^ and flower-like crystal morphologies in copper metal–organic framework crystals.^37^

**Figure 6:**
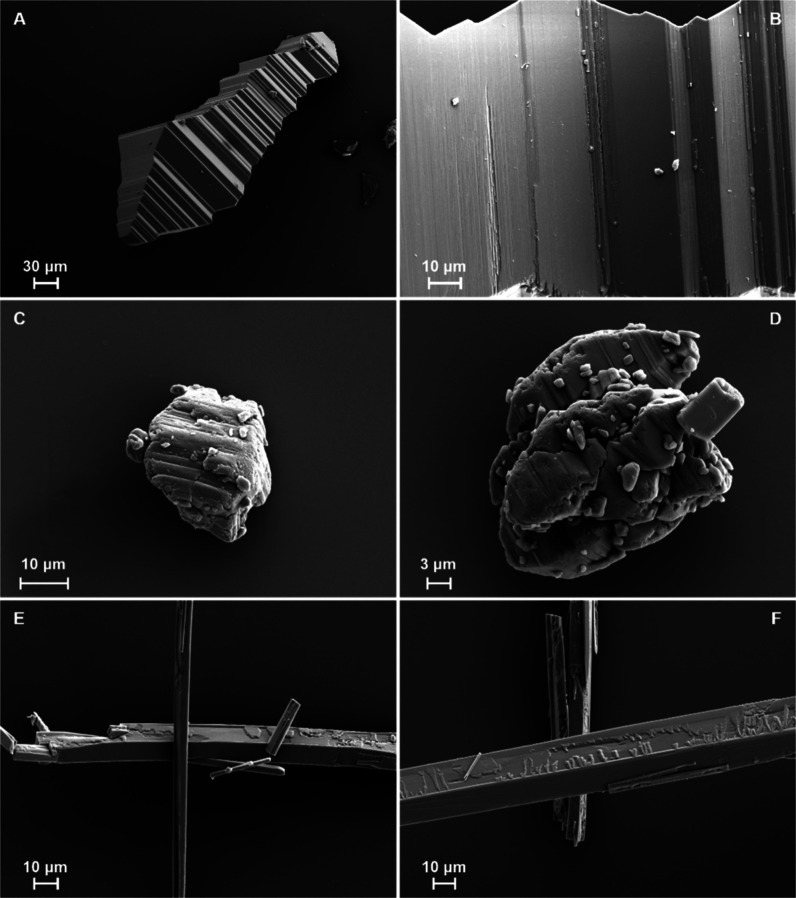
SEM micrographs of TeDi at different magnifications. (A) A single FI accordion crystal with (B) showing the thin layers of the parallel growth in greater detail. (C,D) show the as-supplied FI powder with a similar layered appearance to the FI crystals. (E,F) show the macrosteps on specific FII needle faces.

For FII TeDi, the SEM micrographs revealed macrosteps on specific faces of the needles when isolated from solution (Figure 6E,F). These macrosteps can be attributed to 2D nucleation and growth of FII monolayers on the facet. A handful of examples of polymorphic systems have described the epitaxial nucleation of a metastable form on the surface of a more stable form in steroidal systems^38–40^ and composite crystals containing sulfathiazole polymorphs;^41^ however, we cannot definitively suggest this in the case of TeDi. Additional SEM micrographs for the FI powder, FI accordions, and FII needles can be found in Figures S2–S4.

### Experimental and Computational Polymorph Stability

When considering Ostwald’s Rule of Stages,^42^ it is difficult to determine which TeDi polymorph grows first in solution (and therefore the metastable form), due to their concomitant nature.^43^ To experimentally investigate the stability relationship of the two polymorphs, differential scanning calorimetry (DSC) and thermogravimetric analysis (TGA) were employed; however, both forms decompose upon heating above 300 °C. The peak decomposition temperature is slightly higher for FI, which may suggest a more stabilizing lattice energy; however, this cannot be quantified (Figure S5). As a result, the solvent-mediated polymorphic transformation method^44^ was utilized to determine the experimental stability of the polymorphs. This involved slurrying a suspension of the polymorph crystals in water on a roller mixer and monitoring the sample visually and by PXRD for 1 day and 1 week. In both cases, only the FI accordion crystals remained, suggesting that they are the thermodynamically stable form (Figure S6).

Experimentally, both polymorphs contain different conformations, where the –CONHNH_2_ groups are slightly twisted from the benzene ring plane (Figure S7). Conformational analysis on the TeDi molecule was carried out by generating and optimizing all possible conformers to determine whether different conformers could be located and lead to the discovery of other TeDi polymorphs. The conformational analysis was performed by systematically sampling the rotational space for all torsion angles of TeDi using Møller–Plesset (MP2) and hybrid density functional theory (DFT) with the IEF-PCM solvation model applied using water as solvent. Twelve minima of TeDi were located, but only four conformers were thermodynamically favorable, being within 1.1 and 0.3 kJ/mol of each other as computed with MP2/6-311G­(d,p) and PBE0/Def2TZVPP, respectively (Figures S8 and S9). In the four lowest energy conformers, each NH_2_ unit is oriented so that an intramolecular NH···O interaction is present. FII has a conformation similar to that of one of the lowest energy conformers generated computationally. The FI crystal structure contains less favourable –CONHNH_2_ group orientations, which are responsible for the relatively high single-point energy of the isolated conformation, at 9.9 and 16.3 kJ/mol higher in energy compared to FII at MP2/6-311G­(d,p) and PBE0/Def2TZVPP, respectively (Tables S2 and S3). The unusual –CONHNH_2_ group orientations adopted in the crystal of FI are presumably due to the strong and weak intermolecular NH···O interactions. Calculated intermolecular interactions of pairs and their corresponding intermolecular energies can be found in Tables S4 and S5.

In addition to the conformational analysis of TeDi, lattice energy calculations of the polymorphs were undertaken using the single-crystal structures of FI and FII, using periodic and molecular DFT. The relative lattice energies were calculated using hybrid intermolecular (PBE-MBD) and intramolecular models (PBE-TS, PBE-MBD, and B2PLYPD/Def2TZVPP),^45–49^ Tables S6–S8. For FI and FII, the relative lattice energies from the three hybrid models suggest that in addition to having the more stable conformer as described above, FII also has the most thermodynamically stable lattice at 0 K, with the PBE-MBD/B2PLYPD model^50^ predicting a 6.99 kJ/mol energy difference between the two forms (Figure 7). Calculating the Helmholtz free energy on both forms between 0 and 500 K also shows that FII is more stable than FI computationally within that entire temperature range (Figure S10). The greater intermolecular energy for FII can be attributed to the presence of π–π stacking between the aromatic rings, in addition to the extended hydrogen bond network, which further enhances its overall stability computationally.

**Figure 7:**
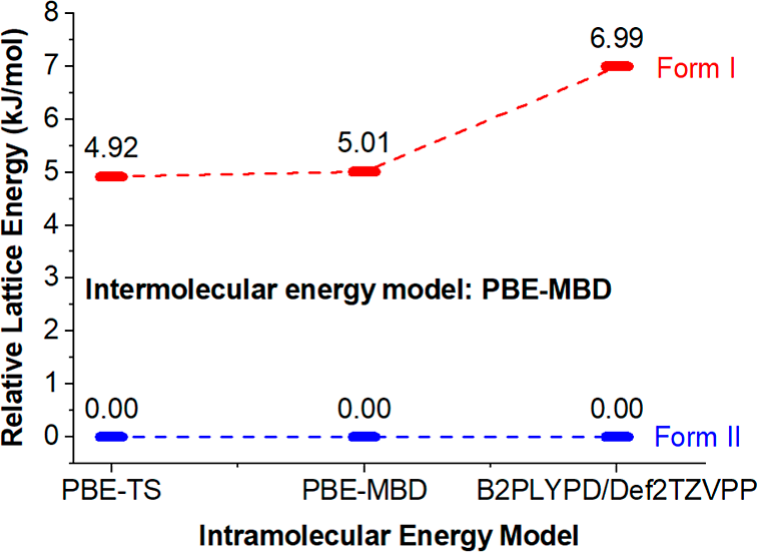
Relative lattice energies of FI and FII as computed with three hybrid DFT-d models.

Given the surprising observation from conformational analysis and DFT-d that FII should be the most stable form, in contrast to experimental observations, crystal structure prediction (CSP) methods were performed to generate and explore the crystal energy landscape of TeDi. The utilized CSP methodology, as described in the method sections of the Supporting Information, accounts for molecular flexibility in both the structure generation^51–54^ and refinement stages^55^ and uses a hybrid ab initio empirical force field. The CSP results are presented in Figure 8 with FI (ranked 96th) and FII (ranked fifth) successfully generated computationally and plotted in red and blue, respectively. The full candidate generation landscape and FI and FII ranking details can be found in Figure S11 and Table S9. These CSP calculations, therefore, once again contrast with the experimental observations and agree with the more accurate DFT-d hybrid methods, where FII was calculated to be more stable than FI (by ∼11 kJ/mol with the CSP model). The landscape identifies a global minimum structure 3.2 kJ/mol more stable than the experimental FII and a structure very similar to FI, ranked as 105th (P105) and within less than 0.5 kJ/mol of FI. An overlay of FI and P105, as calculated with Mercury,^56^ is presented in Figure 9A. Structures FI and P105 are very similar. They are both layered structures with identical packing (Figure 9A), differing only in the orientation of TeDi molecules (related by a 2-fold rotation) every two stacked layers. For every two identical stacking layers, there are two mismatched layers, as shown in Figure 9B. The mismatched layers occur along the twinning plane, as characterized experimentally, hence representing an “ideal” model of a related structure and providing insights into the change in molecular orientation (upon attachment of a new stacking layer), plausibly leading to stacking faults and macroscopic twinning.

**Figure 8:**
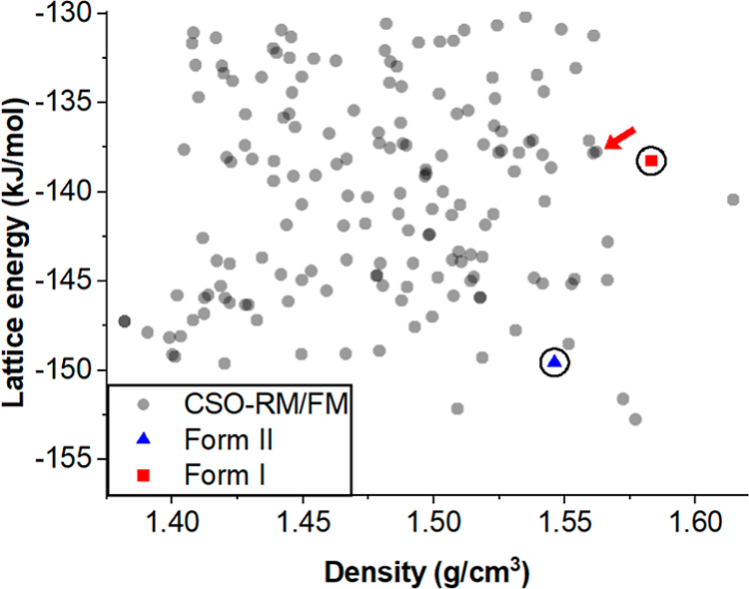
CSP landscape for TeDi. All generated structures are plotted in gray and the generated structures matching experimental FI and FII are highlighted in red and blue, respectively. The structure highlighted with the red arrow (rank 105) is very similar to FI.

**Figure 9:**
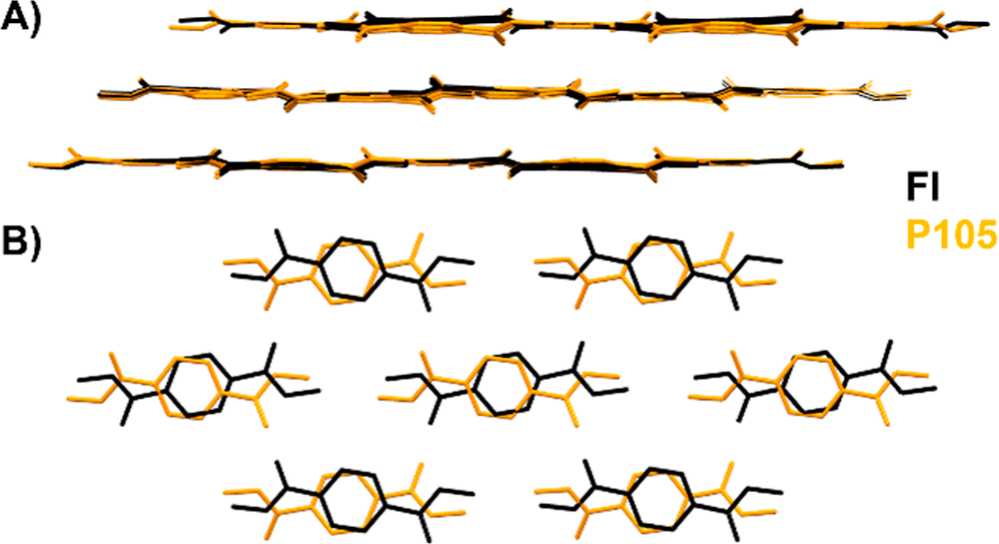
Overlay of structures FI (black) and P105 (orange). (A). The structures are very similar and consist of two matched layers with identical positions for TeDi and two mismatched layers. The overlay of the mismatched layers is shown in (B).

Real crystals, unlike ideal crystals, can be complex, with many types of disorder and defects possible (in this case, stacking faults and twinning), which are extremely difficult to model and could impact the divergence between experiment and computation in this case. To understand the impact of a “mixed” stacking on the overall stability of FI, FI and P105 were combined into an ideal mixed crystal by calculating the ideal entropy of mixing of these two forms using eq 1. The lattice energy for P105 was then calculated using the DFT-d hybrid models, and the overall free energy of FI at room temperature was calculated as a function of mixing, as shown in Figure 10. The mixing between pure FI and P105 is favorable for the overall free energy of FI, with a minimum in free energy at ∼0.4 fraction of P105. These calculations, therefore, suggest that the stacking faults should be favorable and lead to a mixed structure with ∼40% of P105 and 60% of FI, in excellent agreement with the X-ray derived twin ratios. However, the maximum stabilization gained on this mixing is only −0.6 kJ/mol, suggesting that this effect is still not enough to reverse the predicted order of stabilities between FI and FII of 7 kJ/mol.

1 $${\Delta S}_{mix}=-R\left(x_{FI}\ln x_{FI}+x_{P105}\ln x_{P105}\right)$$

**Figure 10:**
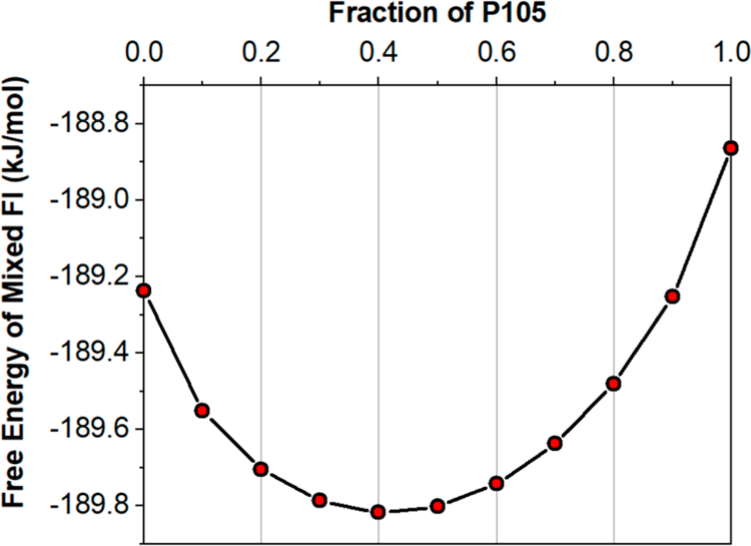
Free energy of FI at room temperature calculated as a function of mixing with the P105 structure.

From an intermolecular interaction perspective, in the case of twinned saccharin crystals, an interfacial hydrogen bond was found to be a driving force for rapid twinning,^57^ as twinning will only occur when the intermolecular interactions across the twin boundary are energetically competitive with those present in a non-twinned crystal.^58^ It could be postulated that for TeDi, increasingly favorable intermolecular interactions (e.g., shorter hydrogen bond and/or aromatic stacking distances) occur at the twin domain interface between the multiple alternating twin layers of the FI accordions, compared to weaker interactions between molecular layers of the same twin domain; however, this is yet to been proven.

### Extended Polymorph Search

The CSP landscape highlights numerous potential undiscovered TeDi polymorphs. In pursuit of additional polymorphs (and due to the success of high-pressure polymorphs of oxalyl dihydrazide), FI and FII were submitted for high-throughput encapsulated nanodroplet crystallization screening (ENaCt)^59^ and high-pressure crystallography experiments; however, no new TeDi polymorphs or further phase changes were observed. An alternative approach to discovering unobserved experimental polymorphs or even to separate concomitant polymorphs is through supramolecular gelation. A low-molecular-weight-gelator (LMWG) known for over 100 years is 1,3:2,4-dibenzylidene-_D_-sorbitol (DBS) and it has been well-characterized.^60–62^ In spite of its long history and extensive study, the self-assembly mode of DBS remains somewhat in doubt, with a variety of reports highlighting different interactions as being fundamental to this process.^63–70^ Taking an overview of these different studies might indicate that in organic solvents, DBS primarily assembles as a result of hydrogen bond interactions between the sorbitol “bodies”, while in more polar solvents, a solvophobic contribution from the aromatic “wings” can contribute, although the extent of aromatic “stacking” remains contested. The limited aqueous solubility of DBS has led to its chemical modification, specifically with the addition of a carboxylic acid group^71^ and *bis*(acylhydrazide) groups^72^ (DBS-CONHNH_2_, Figure 11). The DBS-CONHNH_2_ gelator displays improved water gelation over a greater pH range compared to its carboxylic acid equivalent.^72,73^ Interestingly, we noted that the two phenylene acylhydrazide groups of DBS-CONHNH_2_ are structurally identical to TeDi, suggesting that DBS-CONHNH_2_ may be a very suitable supramolecular TeDi mimetic gelator.

**Figure 11:**
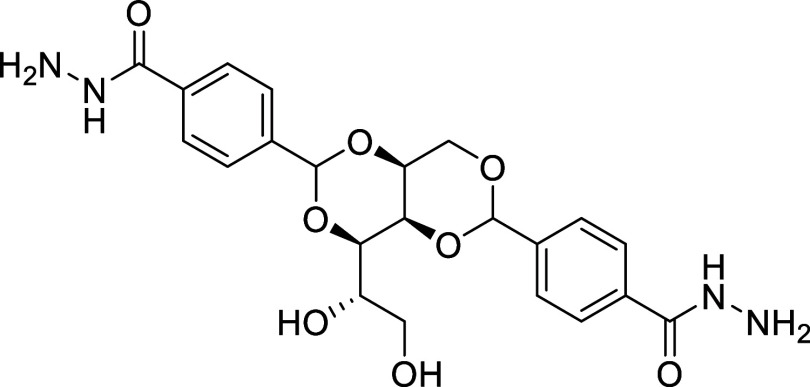
Chemical structure of DBS-CONHNH_2_ gelator.

The heating and subsequent cooling of different concentrations and supersaturations of TeDi powder in the presence of DBS-CONHNH_2_ in water at 0.4 wt %/vol produced stable translucent hydrogels containing only TeDi FII crystals, as evidenced by PXRD studies of the dry gel and crystals (Figure S12). Additionally, the FII crystals also remain in the gel for months, in contrast to those in solution which readily convert to FI. When the same TeDi concentrations, supersaturation levels, and crystallization conditions are applied without the gelator, the concomitant crystallization of FI and FII is observed. This is an interesting example of a supramolecular gel capable of controlling the crystallization of a polymorphic system within a self-assembled network.

To investigate the selective crystallization of FII in the gel further, we studied the solid-state behavior of the gelator itself. It is worth noting that LMWGs are notoriously difficult to crystallize since they typically form unidirectional interactions and hence self-assemble into nanofibers without significant three-dimensional crystal growth.^60^ The structures of different DBS gelators, including DBS-CONHNH_2_, have been predicted computationally but not previously experimentally realized.^74^ With the use of 3D electron diffraction, crystal structures can be determined from crystalline powders without the need to grow large single crystals. In this way, we successfully structurally characterized DBS-CONHNH_2_. The LMWG adopts a butterfly-like conformation, with the sorbitol hydrophilic and chiral “body” and the phenylene *bis*(acylhydrazide) hydrophobic “wings”.^60,65^ Descriptions of the crystal structure and its interactions can be found in the Supporting Information, along with hydrogen bond details (Table S10), and the experimental and calculated PXRD diffractograms, compared to the air-dried gel (Figure S13). The crystal packing of the LMWG exhibits a herringbone pattern analogous to that of the crystal packing of FII (Figure 12). Therefore, we propose that the gelator structure acts as a template to nucleate FII on the gelator fibers, and as a result, the nucleation and growth of FI crystals are inhibited within the gel network.

**Figure 12:**
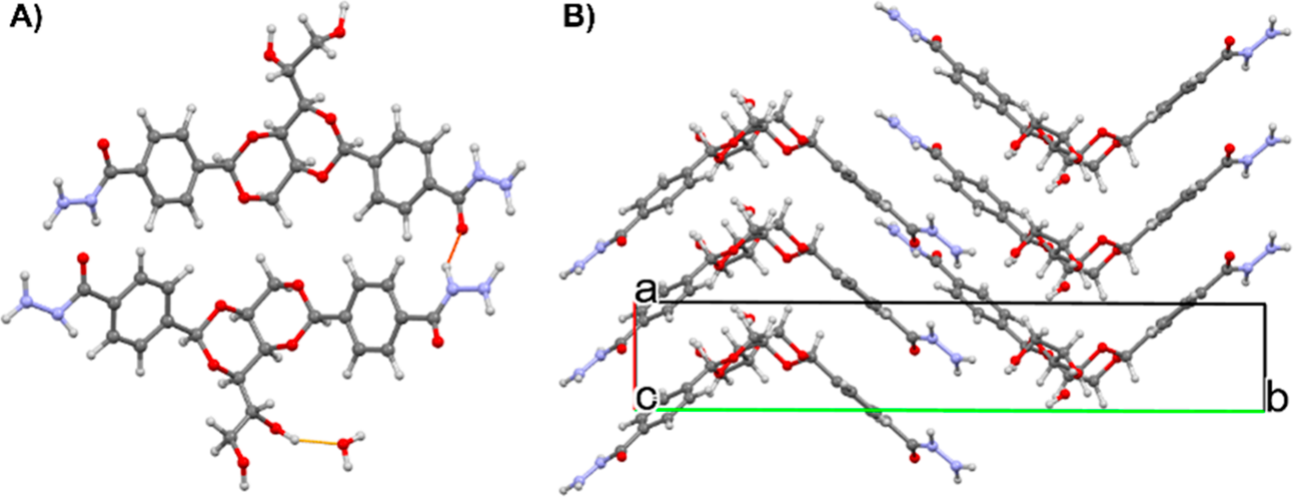
The crystal structure of DBS-CONHNH_2_·0.5H_2_O in the (A) asymmetric unit—hydrogen bonds are colored by interaction distance: short (yellow), mid (red), and long (blue) and (B) the most favorable stacking arrangement of the sugar units and aromatic interactions (hydrogen bonds removed for clarity).

## Conclusions

Polymorphism is a system-dependent phenomenon and is not always simple to understand and control, especially when the crystallization of two polymorphs is concomitant. The heating and fast cooling of TeDi powder provide two polymorphs, FI and FII. FI has a novel and unprecedented accordion crystal morphology not seen before in organic systems and is the most experimentally stable form, compared to FII needles that disappear in solution after 20 h. Employed computational methods (conformer generation and analyses, lattice energy calculations, and crystal structure prediction calculations) all determine FII as the most thermodynamically stable form. As twinning or stacking faults were not accounted for due to the nature of the calculations, an ideal mixing model was employed to compute the entropy of mixing between FI and a calculated twin domain structure; however, this effect still did not reverse the predicted stability order. Accurate models are still needed for complex polymorphic systems with disorder and twinning, especially as some polymorphic systems lie within a very small energy range.

A way to isolate FII from the concomitant solution crystallization of TeDi is through crystallization with a supramolecular mimetic gelator. The solid-state behavior of the DBS-CONHNH_2_ gelator has been studied for the first time, with electron diffraction of the gelator powder revealing a herringbone crystal packing of the molecules, dominated by the stacking of the sugar units and aromatic rings. The packing and interactions of the gelator are similar to the crystal structure of FII, which enables the gel fibers to template the growth of only FII crystals.

In summary, experimental crystallization can lead to unexpected outcomes with intriguing details, particularly defects and disorder (which result in a crystal morphology previously unseen in organic crystals), highlighting the compelling case where real crystal behavior is not consistent with current high-level computational methods and emphasizes the key areas for development at the forefront of advanced computational methods.

## Supplementary Material



## Notes

Additional experimental and computational details, materials, and methods; crystallographic information files for all crystal structures discussed in the manuscript; and crystal structure prediction generation and refinement files (PDF)

## #

Stefanos Konstantinopoulos is currently at Department of Chemical Engineering, Imperial College London, South Kensington, London, SW7 2AZ, UK

## Abbreviations

TeDi: terephthalic dihydrazide
FI: form I
FII: form II
LMWG: low-molecular-weight-gelator
CSD: Cambridge Structural Database
SEM: scanning electron microscopy
PXRD: powder X-ray diffraction
DSC: differential scanning calorimetry
TGA: thermogravimetric analysis
MP2: Møller–Plesset
DFT: density functional theory
DFT-d: dispersion-corrected density functional theory
CSP: crystal structure prediction
ENaCt: encapsulated nanodroplet crystallization
