# Supplementary material for: When Reality Defies Prediction: Polymorphism, Twinning, and Accordion Crystals
Source: J Am Chem Soc. 2026 Jan 27;148(5):5774–82. doi: 10.1021/jacs.5c22213 (PMC12903866; doi:10.1021/jacs.5c22213)
Supplement: Supplementary file 1 [file ja5c22213_si_001.pdf]

When Reality Defies Prediction: Polymorphism, Twinning, and Accordion Crystals

Amy V. Hall,* Alice C. Taylor, Natalie E. Pridmore, Aurora J. Cruz-Cabeza, David K. Smith, Niccolò Cosottini, Mark A. Fox, Amrita Chattopadhyay, Stefanos Konstantinopoulos, Daniel N. Rainer, Simon J. Coles, Nicholas Blagden, Qi Zhang, Leon Bowen, Toby J. Blundell.

Email: amy.v.hall@durham.ac.uk

Supporting Information

Contents:

1. SI Figures S1-13 and Tables S1-10.
2. All experimental methods that detail the work undertaken and analyzed (hydrogen bond definition, instrument details, crystallization details).
3. All computational methods used in the manuscript (conformer calculations and analyses, lattice energy calculations, and crystal structure prediction details).
4. All references cited in the SI.

1. SI Figures and Tables

Table S1. Hydrogen Donor atom (D), hydrogen atom (H), acceptor atom (A) and their distances and angles with estimated standard deviations for TeDi FI accordions and FII needles. The values without estimated standard deviations were refined using a riding model.

	**D H A**	**D-A/ Å**	**D-H-A/ °**
**FI**	N1 H1 O2	2.851(5)	171.4
	N3 H3 O1	2.901(5)	160.2
	N4 H4A O1	3.143(5)	153.8
	N4 H4B O2	3.105(5)	167.4
**FII**	N2 H2A O1	2.978(3)	123(3)
	N2 H2B O1	2.959(3)	174(3)
	N1 H1 N2	2.914(4)	157(3)

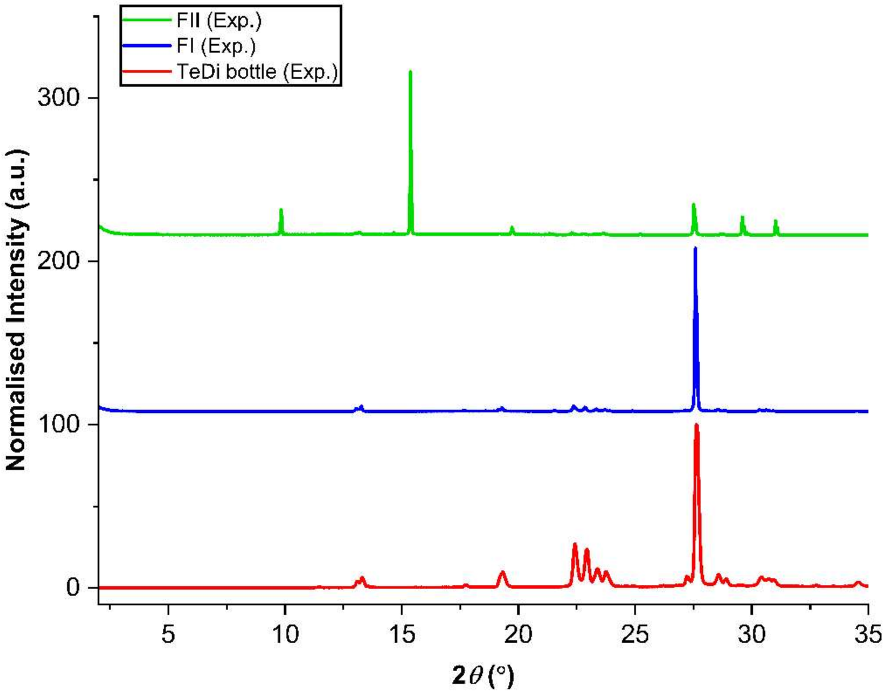

Figure S1. The experimental powder diffractograms of TeDi as supplied powder (TeDi bottle), FI, and FII. The diffractograms highlight that the TeDi as-supplied powder is in agreement with the pattern of FI only.

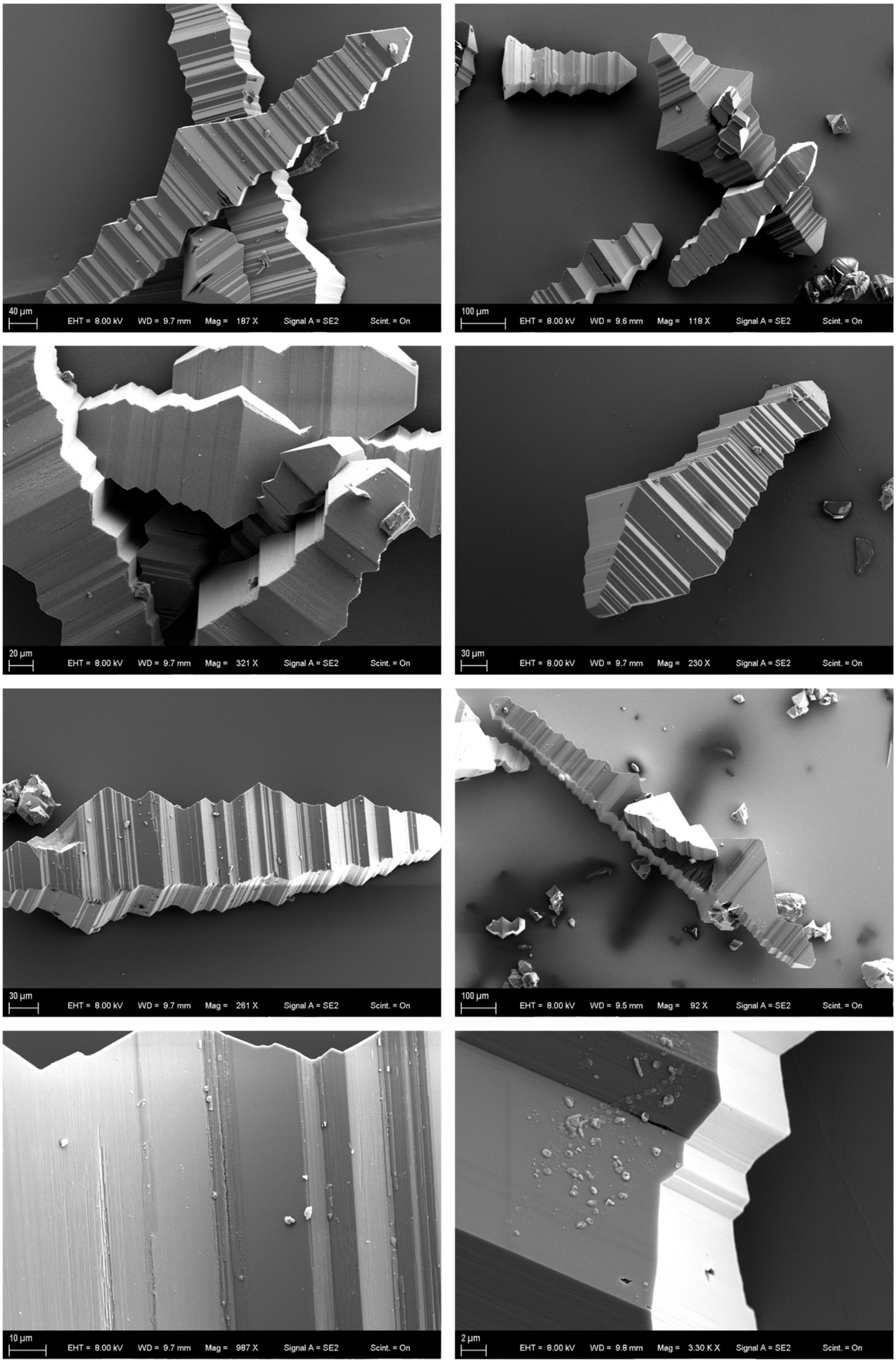

Figure S2. SEM micrographs of the FI accordion crystals at different magnifications.

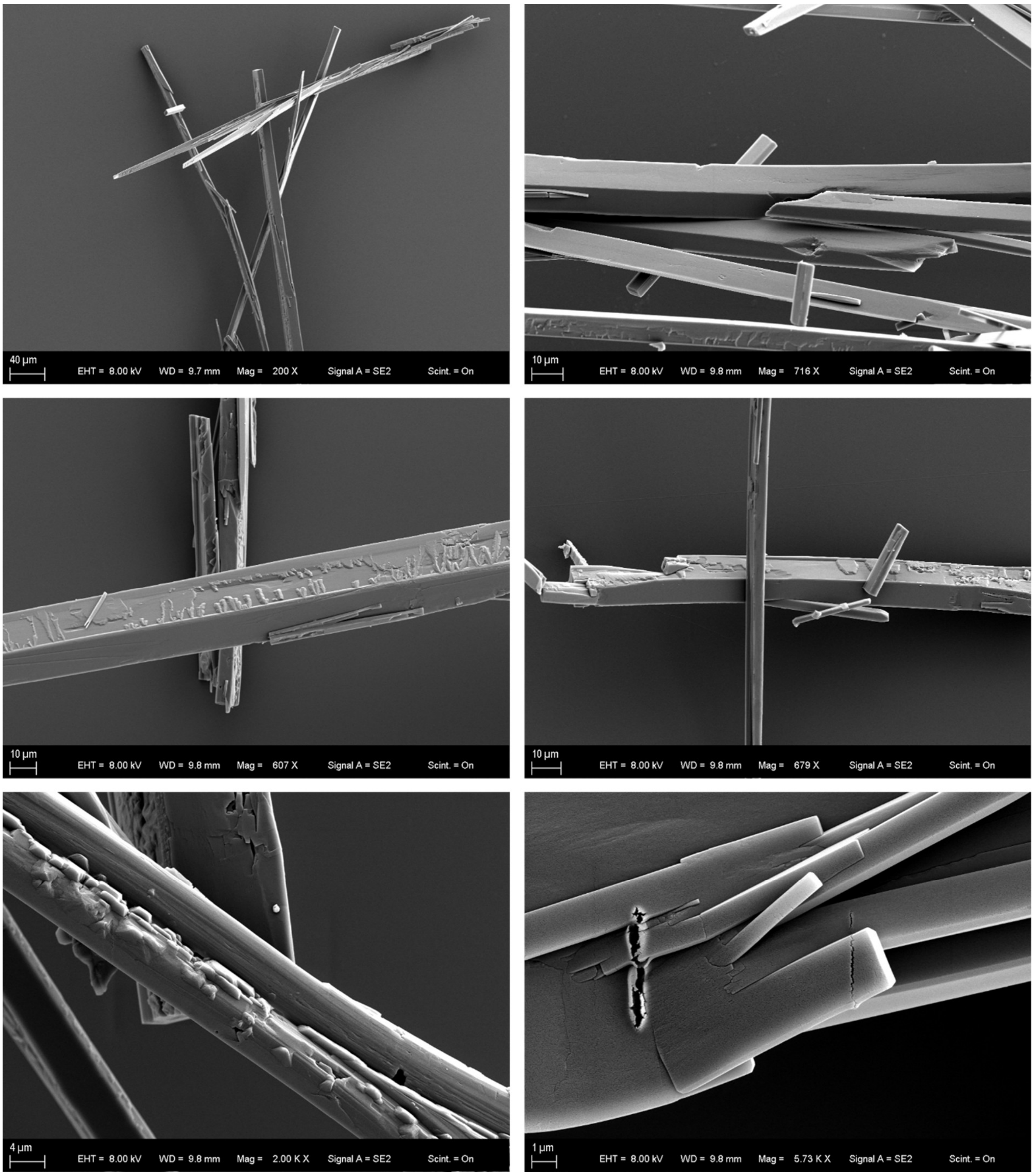

Figure S3. SEM micrographs of the FII needle crystals at different magnifications.

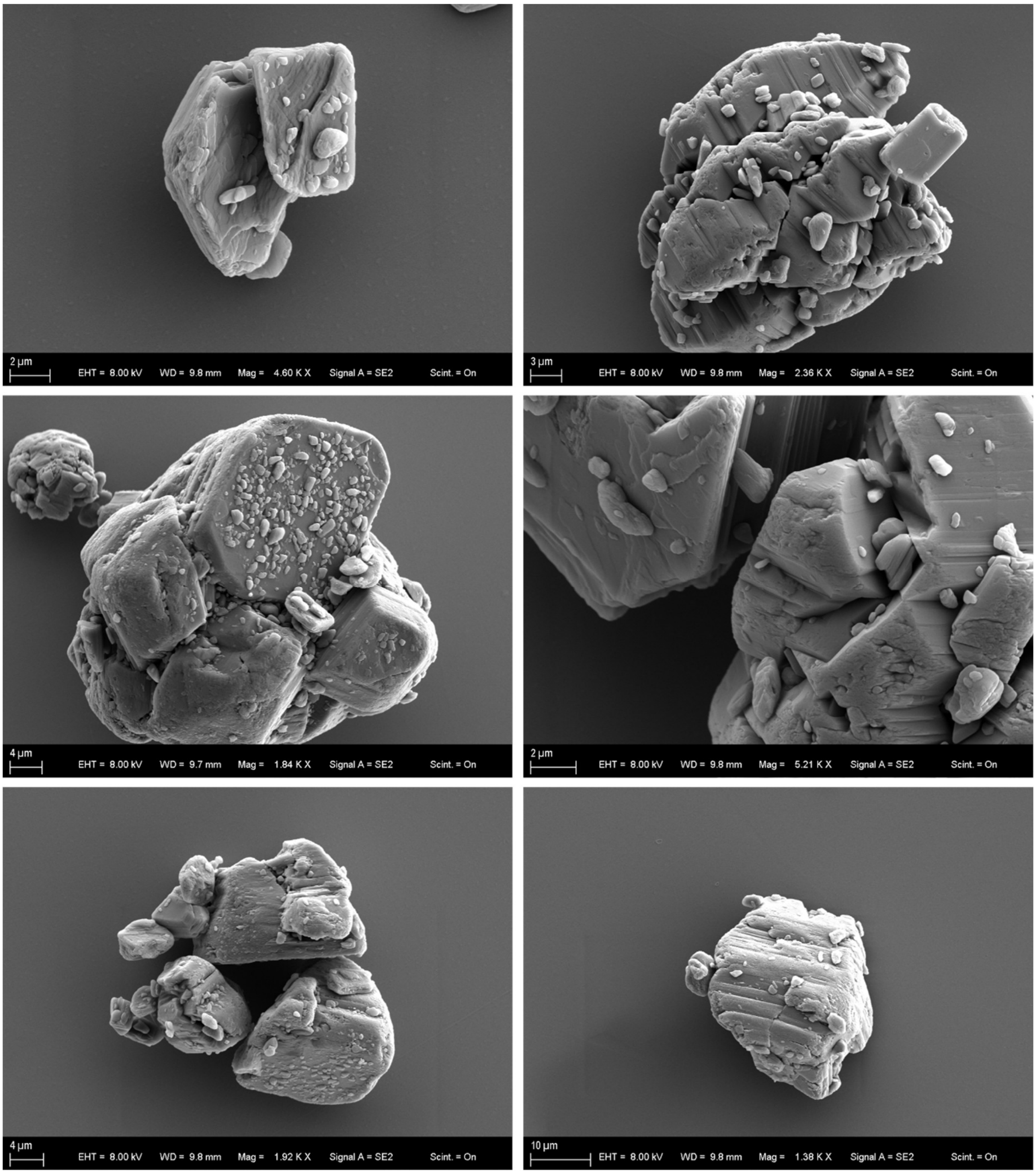

Figure S4. SEM micrographs of the FI powder as supplied at different magnifications.

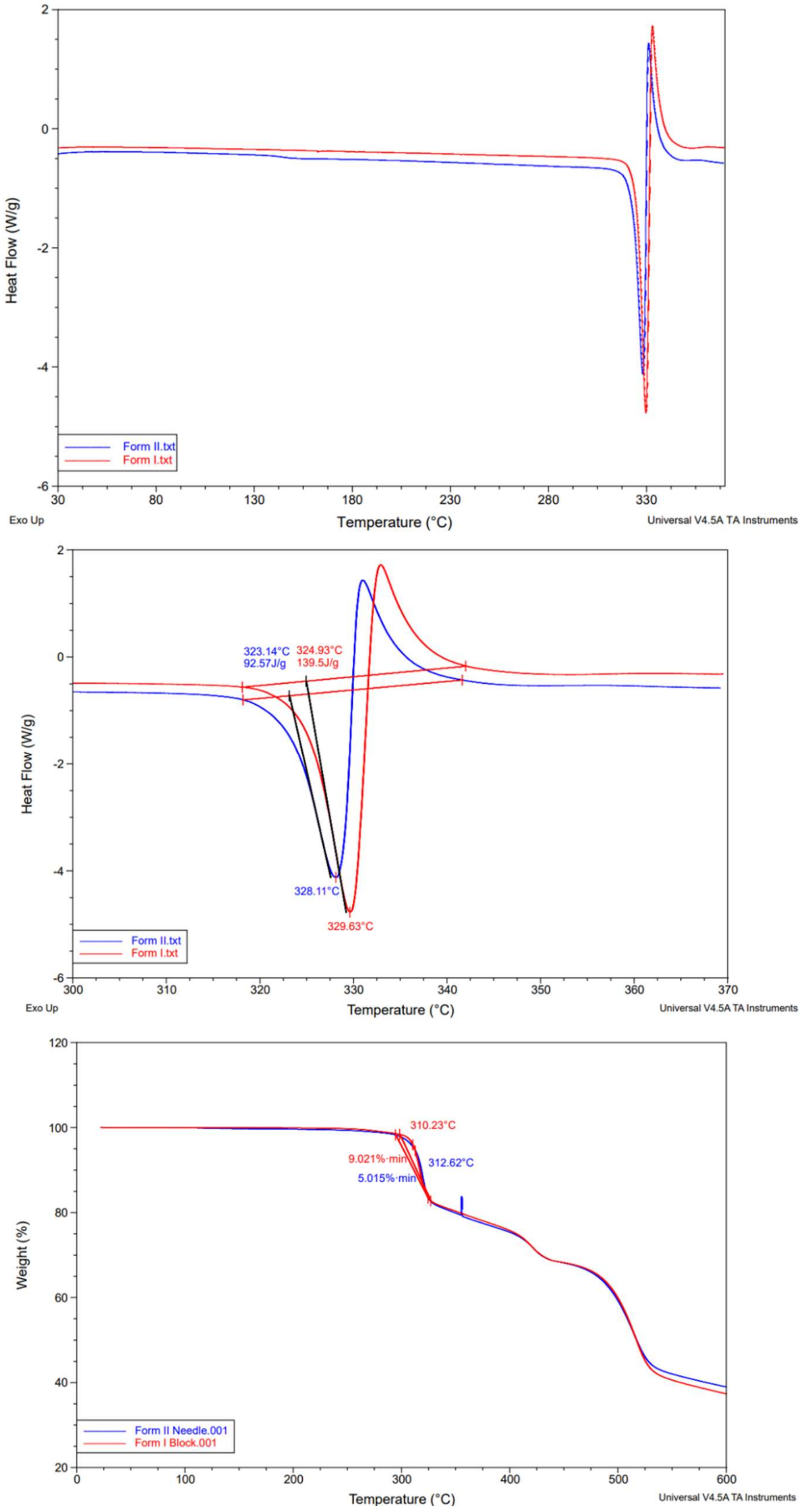

Figure S5. The DSC thermogram of the FI and FII TeDi crystals (top) and a zoomed region to show the DSC heating events clearly for the two polymorphs (middle), and the TGA (bottom) thermogram of FI and FII crystals. DSC and TGA show melt-decompositions after 300 °C.

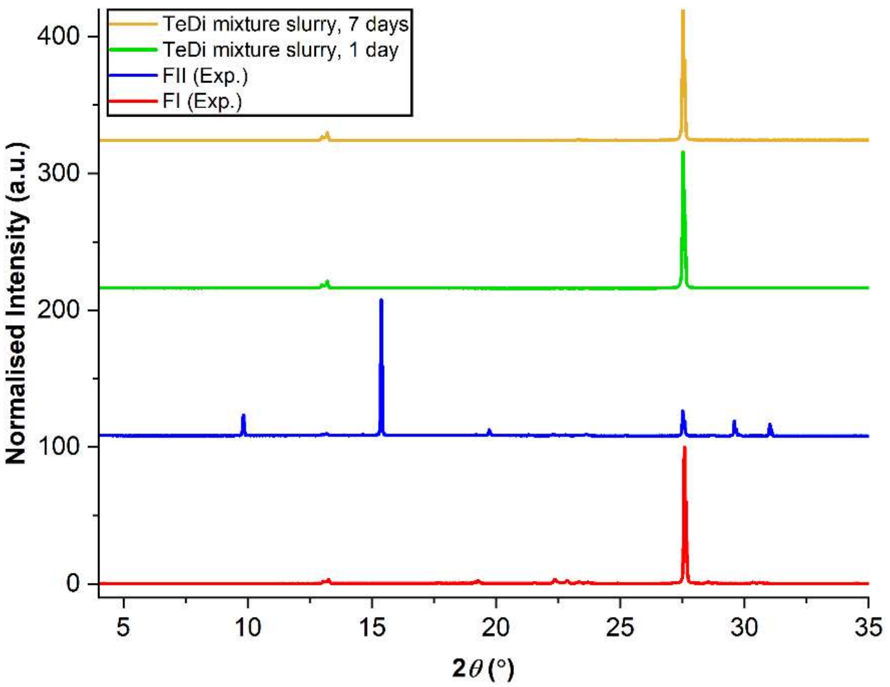

Figure S6. The experimental PXRD diffractograms of TeDi polymorphs and the combined TeDi polymorphs slurried in water after one day and 7 days.

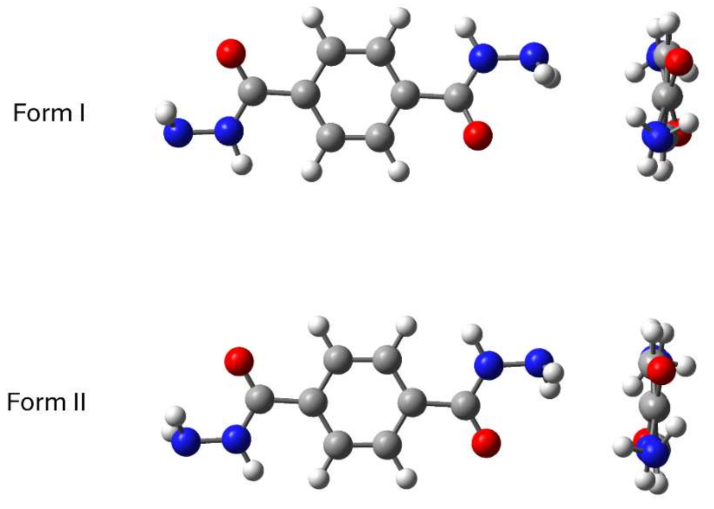

Figure S7. Plan and side views of TeDi conformations in FI and FII.

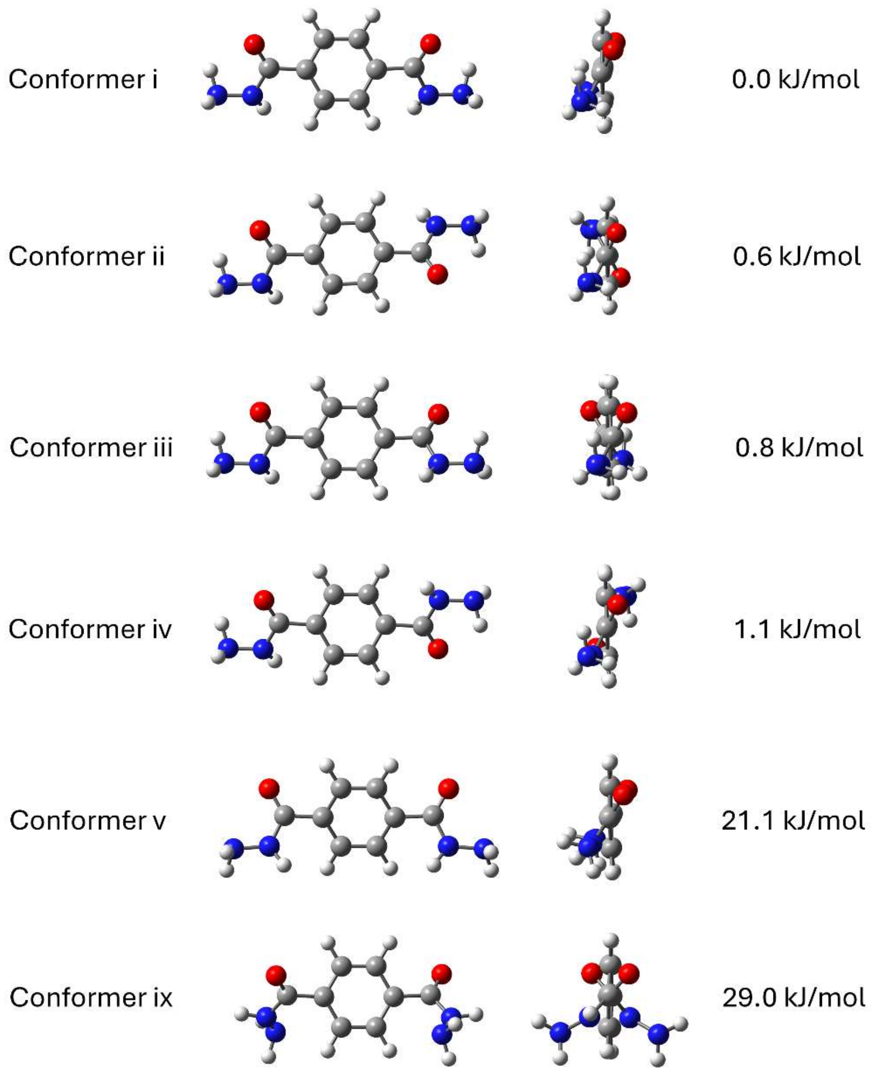

Figure S8. Plan and side views of TeDi conformers from geometry optimizations with relative Gibbs free energies, calculated using MP2/6-311G(d,p). Conformers vi, vii and viii contain one $CONHNH_2$ group in different orientations to conformer v. Conformers ix-xii contain NH_2 groups in different orientations to the corresponding conformers i-iv.

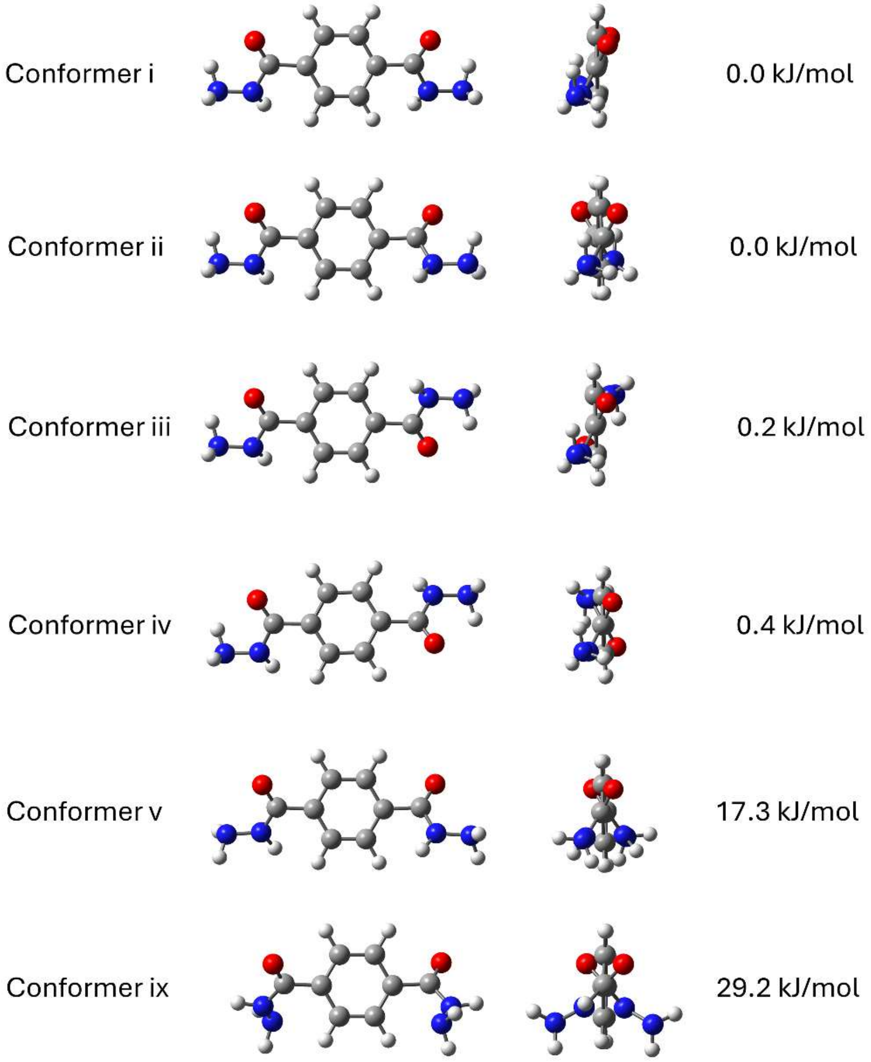

Figure S9. Plan and side views of TeDi conformers from geometry optimizations with relative Gibbs free energies, calculated using PBE0/Def2TZVPP. Conformers vi, vii and viii contain one $CONHNH_2$ group in different orientations to conformer v. Conformers ix-xii contain NH_2 groups in different orientations to the corresponding conformers i-iv.

Table S2. Selected torsion angles and relative energies of optimized TeDi conformers, calculated by MP2/6-311G(d,p) with IEF-PCM solvation model using water as solvent. The X-ray structures of FI and FII were not optimized. FII resembles conformer iv.

Conformer	OC…CO torsion angle (°)	Acute HN-NH torsion angle (°)	Relative electronic energy (kJ/mol)	Relative Gibbs free energy (kJ/mol)
i	0.0	117.8	0.2	0.0
ii	118.1	117.5	0.0	0.6
iii	62.3	117.4	0.2	0.8
iv	180.0	117.7	0.2	1.1
v	0.0	54.7	20.0	21.1
vi	177.5	54.7	20.1	21.2
vii	110.7	54.6	19.9	21.3
viii	68.1	54.5	20.1	21.7
ix	85.5	47.7	26.4	29.0
x	0.0	47.8	26.6	29.7
xi	94.5	47.5	26.2	30.2
xii	180.0	47.6	26.5	30.8
FI	147.6	119.9	32.6	
FII	180.0	97.4	22.7	

Table S3. Selected torsion angles and relative energies of optimized TeDi conformers, calculated by PBE0/Def2TZVPP with IEF-PCM solvation (water as solvent) and GD3BJ dispersion models applied. The X-ray structures of FI and FII were not optimized. FII resembles conformer iii.

Conformer	OC…CO torsion angle (°)	Acute HN-NH torsion angle (°)	Relative electronic energy (kJ/mol)	Relative Gibbs free energy (kJ/mol)
i	0.0	122.9	0.1	0.0
ii	56.1	122.5	0.2	0.0
iii	180.0	121.7	0.1	0.1
iv	123.3	121.8	0.0	0.3
v	62.3	58.0	16.8	18.1
vi	180.0	58.6	16.7	18.1
vii	0.0	58.1	16.6	18.9
viii	119.8	58.5	16.5	19.4
ix	89.6	54.2	27.6	30.0
x	90.3	54.0	27.4	31.2
xi	0.1	54.1	27.5	31.7
xii	180.0	54.0	27.6	32.5
FI	147.6	119.9	27.9	
FII	180.0	97.4	12.1	

Table S4. Intermolecular interaction energies and type of favorable interactions for each interacting pair of molecules (dimer) in the crystal structures of Forms I and II, calculated using MP2/6-311G(d,p)//IEF-PCM(water).

Interacting pair of molecules	Intermolecular interaction energy (kJ/mol)	N…O distance in N-H…O interaction (Å)	N…N distance in N-H…N interaction (Å)	Stacking interaction	Number of identical pairs in a single molecule
FI					
a	31.5	2.958, 2.979			4
b	27.5		2.914		4
c	58.8			Yes	2
d	18.1			Yes	2
FII					
a	39.8	2.869			2
b	42.9	2.884			2
c	26.9		3.085		2
d	8.7		2.982		2
e	35.4			Yes	2
f	45.7			Yes	2
g	44.4			Yes	2

Table S5. Intermolecular interaction energies and type of favorable interactions for each interacting pair of molecules in the crystal structures of pure Forms I and II, calculated using PBE0/Def2TZVPP//IEF-PCM(water)//GD3BJ.

Interacting pair of molecules	Intermolecular interaction energy (kJ/mol)	N…O distance in N-H…O interaction (Å)	N…N distance in N-H…N interaction (Å)	Stacking interaction	Number of identical pairs in a single molecule
FI					
a	26.5	2.958, 2.979			4
b	22.9		2.914		4
c	36.6			Yes	2
d	12.5			Yes	2
FII					
a	31.8	2.869			2
b	35.1	2.884			2
c	23.9		3.085		2
d	5.5		2.982		2
e	25.2			Yes	2
f	34.1			Yes	2
g	30.8			Yes	2

Table S6. Energetics in kJ/mol with the intramolecular model PBE-TS for FI and FII and intermolecular model PBE-TS-MBD.

System	Conformational adjustment energy (difference)	Conformational change energy	Total intramolecular energy (difference)	Intermolecular energy, PBE-TS-MBD (difference)	Lattice energy (difference)
FI	17.32 (4.25)	0.00	17.32 (4.04)	–213.37 (0.89)	–196.06 (4.92)
FII	13.07 (0.00)	0.21	13.28 (0.00)	–214.26 (0.00)	–200.98 (0.00)

Table S7. Energetics in kJ/mol with the intramolecular model PBE-TS-MBD for FI and FII and intermolecular model PBE-TS-MBD.

System	Conformational adjustment energy (difference)	Conformational change energy	Total intramolecular energy (difference)	Intermolecular energy, PBE-TS-MBD (difference)	Lattice energy (difference)
FI	16.99 (4.17)	0.00	16.99 (4.13)	–213.37 (0.89)	–196.39 (5.01)
FII	12.82 (0.00)	0.04	12.86 (0.00)	–214.26 (0.00)	–201.40 (0.00)

Table S8. Energetics in kJ/mol with the intramolecular model B2PLYPD/Def2TZVPP for FI and FII and intermolecular model PBE-TS-MBD.

System	Conformational adjustment energy (difference)	Conformational change energy (difference)	Total intramolecular energy (difference)	Intermolecular energy, PBE-TS-MBD (difference)	Lattice energy (difference)
FI	21.60 (6.76)	0.00	21.60 (6.10)	–213.37 (0.89)	–191.77 (6.99)
FII	14.84 (0.00)	0.66	15.50 (0.00)	–214.26 (0.00)	–198.76 (0.00)

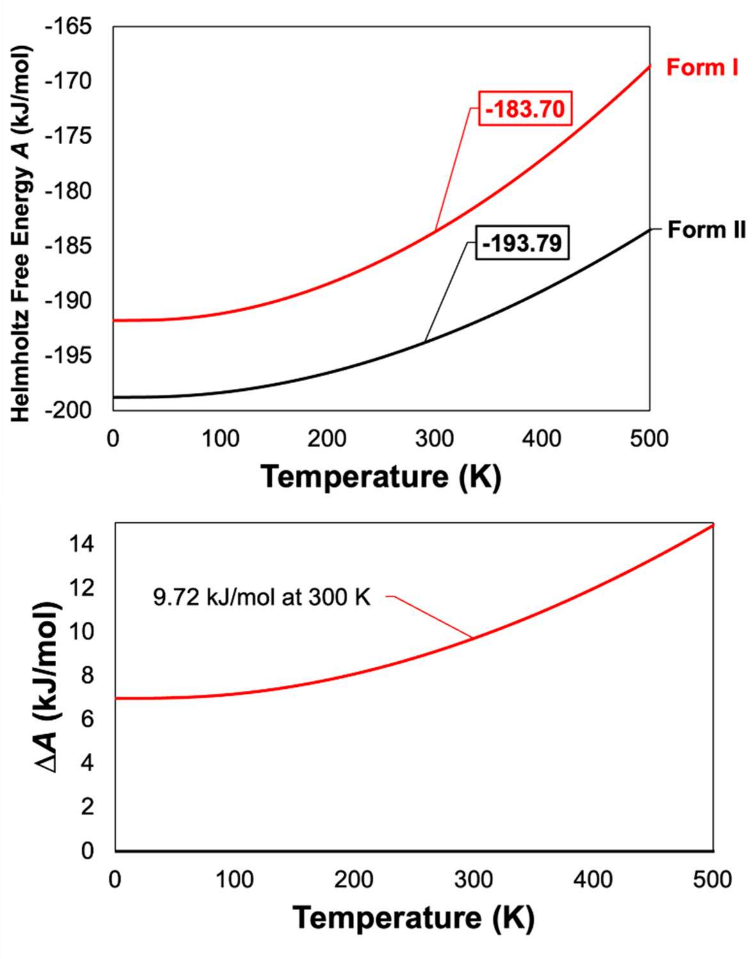

Figure S10. The Helmholtz free energy of both forms at 0 to 500 K, with a free energy difference of 9.72 kJ/mol at 300 K.

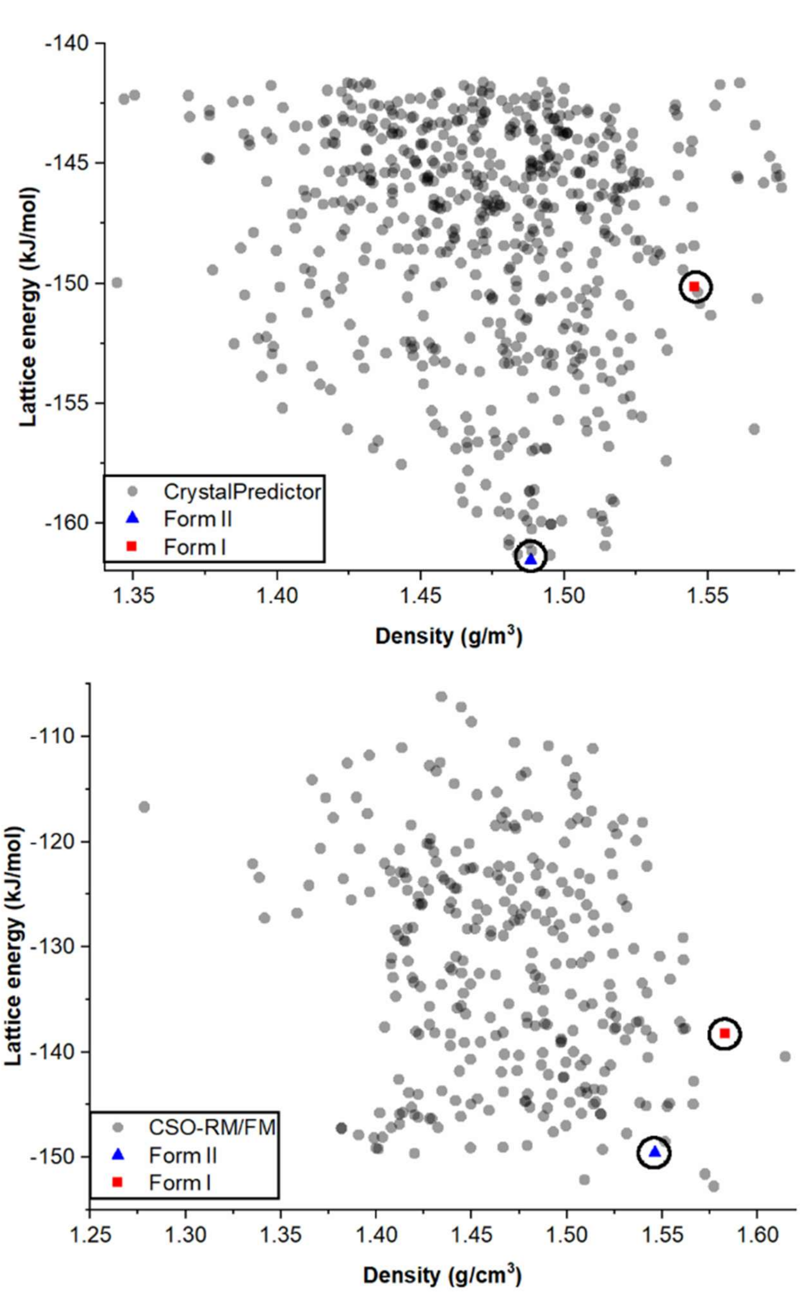

Figure S11. Crystal Structure Prediction landscapes, each dot representing a minimized crystal structure. The full CrystalPredictor landscape (top) and the full CSO-RM/FM landscape (bottom). Theoretical structures that match experimental TeDi polymorphs are represented as a blue triangle for FII, and a red square for FI.

Table S9. Lattice energy, density, geometry similarity, and rank of the theoretical structures in the CrystalPredictor and CSO-RM/FM landscapes, matching the experimental forms of TeDi.

	Lattice energy (kJ/mol)	Relative lattice energy (kJ/mol)	Density (g/cm^3)	RMSD$_{20}$ (Å)	Rank
**Crystal Predictor**					
FI	−150.168	11.373	1.546	0.484	176
FII	−161.541	0.000	1.488	0.545	1
**CSO-RM/FM**					
FI	−138.249	11.338	1.583	0.141	96
FII	−149.587	0.000	1.546	0.440	5

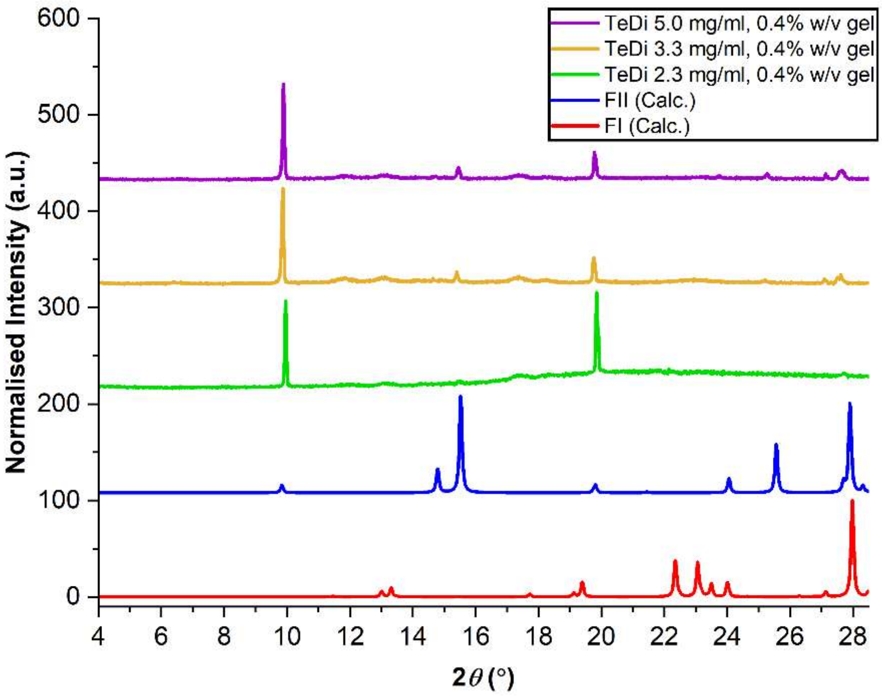

Figure S12. The calculated PXRD diffractograms of TeDi polymorphs and the experimental patterns of TeDi crystallizations in DBS-CONHNH$_2$ gels at different TeDi concentrations. Peaks with amorphous character can be attributed to the presence of the dried gel fibers within the samples.

Table S10. Hydrogen bond information for DBS-CONHNH$_2$. ^{1}2-x,1/2+y,1-z; ^{2}1+x,+y,+z; ^{3}1-x,-1/2+y,1-z; ^{4}-x,1/2+y,-z; ^{5}-1+x,+y,+z; ^{6}1-x,1/2+y,-z; ^{7}-1+x,+y,-1+z. Positions for hydrogen atoms H103, H20a, H20b, H20d, H20f, and H1Wa are geometrically constrained and their D-H distances are kept fixed at published bond lengths from neutron diffraction.[1] Distance restraints (instead of constraints) based on published neutron diffraction data[1] with default standard deviation of 0.02 Å were placed on D-H and H-A distances on hydrogens H10a (1.015; 1.945 Å), H10b (1.015; 1.8 Å), H102 (1.027; 1.85 Å), and H202 (1.027; 1.8 Å).

D H A	d(D-H)/Å	d(H-A)/Å	d(D-A)/Å	D-H-A/deg
N104 H10a O105^1	1.021(16)	1.94(2)	2.870(16)	151(4)
N104 H10b O201	1.046(16)	1.832(17)	2.807(14)	153(3)
N103 H103 O201^2	1.0270	1.997(15)	2.943(15)	151.9(4)
N102 H102 O108^3	1.042(16)	1.87(3)	2.879(16)	163(5)
O204 H20a O1W	0.9830	1.82(3)	2.77(2)	161(6)
N203 H20b O101	1.0270	2.019(16)	2.960(16)	151.2(5)
N202 H202 O208^4	1.021(18)	1.85(3)	2.845(17)	165(5)
N204 H20d O101^5	1.0110	2.053(17)	2.975(16)	150.6(6)
N201 H20f O1W^6	1.0110	2.06(3)	2.97(2)	148(3)
O1W H1Wa O104^7	0.8700	2.21(4)	3.05(2)	161(10)

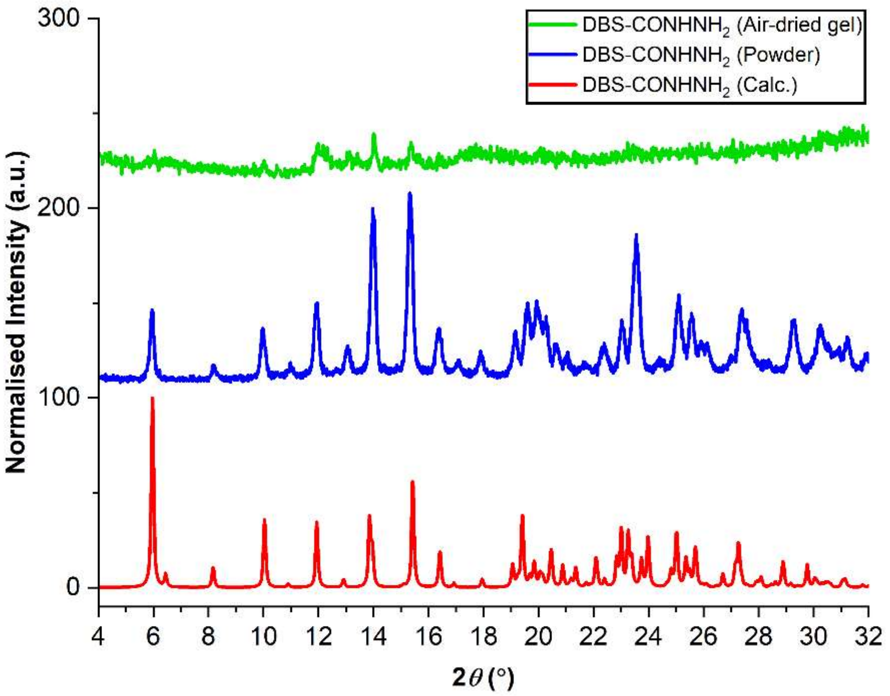

Figure S13. The PXRD diffractograms of the calculated structure of DBS-CONHNH$_2$ from single-crystal XRD data, the experimental DBS-CONHNH$_2$ powder, and the air-dried gel of DBS-CONHNH$_2$. The calculated and experimental powder diffractograms are highly comparable. The air-dried gelator pattern has peaks in agreement with the gelator powder and calculated structure; however, it is significantly less crystalline as expected.

2. Experimental Methods

Hydrogen bond definition

Hydrogen bond contacts were calculated and defined as a hydrogen bond donor (D = O, N, or S), a polarized hydrogen atom and a hydrogen bond acceptor (A = O, N, or S), where the distance between H···A is a minimum distance equaling the sum of van der Waals radii minus 5.00 Å, with a D-H···A angle larger than 120°.

Instrumentation

Thermal analysis was conducted on FI and FII crystals using differential scanning calorimetry (DSC) on a DSC2500 (Thermal Analysis Instruments – Waters LLC) and thermogravimetric analysis (TGA) on a TA Q500. Both methods utilize Universal V4.5A TA Instruments software for data analysis. DSC specifications: approx. 1 mg of the sample was exposed to a heating rate of 5 °C/min. from 25 to 360 °C, with a nitrogen flow rate of 50 mL/min, calibrated using an indium standard, with samples accurately weighed (±0.01 mg) into standard aluminum pans. TGA specifications: approx. 7 mg of the sample was placed in a crucible and heated at a rate of 10 °C/min. from 25 to 600 °C, with a nitrogen flow rate of 50 mL/min.

Scanning electron microscopy micrographs of the FI powder, FI accordion crystals, and the FII needles were acquired at the G.J. Russell Electron Microscopy Facility (Durham University) using a Carl Zeiss Sigma 300 VP scanning electron microscope. The instrument was operated in high-pressure mode with an accelerating voltage of 8 kV. Before imaging, samples were sputter-coated with a 20 nm layer of a gold–palladium alloy (60:40) using a Cressington 108 Auto sputter coater.

X-ray powder diffraction of all materials was performed on low background silicon wafer glass slides using a Bruker AXS D8 Advance diffractometer with a Lynxeye Soller PSD Detector, Cu Ka radiation (1.5406 Å) over an angle range of 2θ = 4˚ to ≥ 30˚, a step size of 0.02˚ and a time per step of 0.6˚/s.

X-ray single crystal diffraction data for the FI blocks, FI accordions, and the FII needles have been collected at a temperature of 120.0(2) K using MoKα radiation (λ =0.71073Å) on a Bruker D8Venture with a Photon III MM C14 CPAD detector, IµS-III-microsource, focusing mirrors diffractometer equipped with a Cryostream (Oxford Cryosystems 700+) open-flow nitrogen cryostat. The structure was solved using Olex2[2] with the ShelXT[3] structure solution program using Intrinsic Phasing and refined with the ShelXL[4] or Olex2.refine[5] refinement packages using Least Squares minimization on F^2. All non-hydrogen atoms were refined with anisotropic displacement parameters. Hydrogen atoms were located on the difference map and refined isotropically on a riding model unless otherwise specified.

X-ray single crystal diffraction data for the FI plates were collected at a temperature of 100.0(2) K at the I-19 synchrotron beamline (λ = 0.68890 Å) with a Dectris Pilatus 2M pixel-array photon-counting detector, undulator and graphite monochromator, at the Diamond Light Source, Oxfordshire. The data was processed using Xia2/DIALS[6, 7] and solved using Olex2[2] with the ShelXT[3] structure solution program using Intrinsic Phasing and refined with the ShelXL[4] using Least Squares minimization on F^2. All non-hydrogen atoms were refined with anisotropic displacement parameters. Hydrogen atoms on heteroatoms were found by difference map after all other atoms had been refined. All other hydrogens were placed geometrically and refined using a riding model.

Crystallographic data for the FI non-twinned plates, FI twinned blocks, FI accordions, and FII needles have been deposited with the Cambridge Crystallographic Data Centre (CCDC) with deposition numbers CCDC 2494050, 2494045, 2494051, 2494046, respectively.

Electron diffraction (3D ED) data were collected on a Rigaku Synergy-ED electron diffractometer (LaB_6, 200 kV), equipped with a Rigaku HyPix-ED hybrid pixel array detector. Data were collected in continuous rotation mode (cRED) using a selected area aperture with a diameter of about 2 μm in the image plane. The white solid sample was gently ground between glass slides and a lacey carbon-coated copper TEM grid (200 mesh, Agar Scientific) dabbed in the solid. The grid was mounted on a Gatan Elsa cryogenic holder (model 698) and transferred into the instrument using cryo-transfer at 175(5) K. The holder was left for about 15 minutes in the airlock chamber to sublime potentially present ice-crystals. The grid was surveyed and data collected on a range of particles using CrysAlisPRO (version 1.171.44.78a).[8] All data collections were conducted at 175(5) K.

Datasets were in each case individually indexed and integrated and subsequently merged and scaled together using CrysAlisPRO (version 1.171.44.79a)[8] and SCALE3 ABSPACK implemented therein. Where necessary, frames were rejected during the processing stage due to holder shadowing and similar effects.

The structure was solved using ShelXD[9] and refined using olex2.refine[5] using the Olex2 GUI (version 1.5-ac7-014)[2] using published scattering factors for electrons.[10] The model was refined using the kinematical approximation in the presence of a refined extinction correction parameter to account broadly for dynamical effects. Hydrogen atoms were generally refined geometrically constrained, their distances fixed to published X—H distances from neutron diffraction[1] and using IADPs with a riding model. On hydrogen atoms bound to N102, N104, and N202, the geometry constraints were removed and D—H distances and/or D—H…A distance restraint to ensure sensible hydrogen atom positions.

One free water molecule per asymmetric unit could be modelled (i.e. half a water molecule per molecule of DBS-CONHNH$_2$)), however, more water may be present in the residual void space.

Individual collections had low completeness and data statistics, therefore crystal structure solution and refinement was performed on the merged dataset. However, given the chiral nature of the compound, individual dynamical refinements against the data from each of the three

individual data collections were performed to determine the correct absolute structure. The previously obtained structure model from the merged set was used as a starting point and dynamical refinements were conducted using the olex2.refine N-beam approach.[5] Due to the low completeness and limited resolution, only the EDT parameter was refined, while all other parameters of the models were fixed. Z-scores obtained for all models agree (Z-scores raw (noise-adjusted): 6.25 (14.88), 5.03 (9.59), 7.40 (15.27)) and indicate that the absolute structure of the model obtained through kinematical refinement displays the correct handedness. 3D ED raw data are deposited at www.doi.org/10.5281/zenodo.15692229.

Crystallographic data for the structure of DBS-CONHNH$_2$ have been deposited with the CCDC deposition number 2465455. These data can be obtained free of charge from The Cambridge Crystallographic Data Centre via www.ccdc.cam.ac.uk/data_request/cif.

Encapsulated Nanodroplet Crystallisation (ENaCt) was accessed through the National Crystallography Service. 2 mg of TeDi powder was dissolved in compatible solvents with sonication (DMSO (196 and 398 µl) and DEF (196 µl) only as water required significant heating) and were subsequently dispensed in 100 nL portions via an SPT Labtech Mosquito liquid handling robot into 96-well glass plates (SWISSCI LCP Modular, 100 µm spacer) containing either an appropriate crystallization oil (300 nL) or no oil. For wells containing oil, the oil was dispensed by the liquid handling robot prior to the injection of the stock solution into the oil droplet. The plate was sealed with a glass cover slip (SWISSCI Modular cover glass, 175 µm thickness) and allowed to stand undisturbed at room temperature in the dark. After 14 days, the crystallization wells were assessed visually and by cross-polarized light microscopy for crystal growth, however, no crystals of TeDi had grown.

High-pressure Crystallography was accessed through the National Crystallography Service. Experiments were attempted for the FI blocks and accordions; however, due to the twinned nature of the crystals, analysis was not possible. However, the FII needles were successfully analyzed but no phase transitions were observed, though two compression regimes were observed across the study. The first compression regime was observed between 0–13.7 kbar, where a small negative linear expansion of the *c*-axis from 18.055(5) Å to 18.151(5) Å, whilst the β angle increased from 94.52(5) ° to 96.10(5) °. This was followed by a second compression regime from 13.7–41.5 kbar, where all axes decreased in length (although the *c*-axis decrease was much less pronounced than for *a* and *b*). The β angle continued to increase up to 97.35(8) ° at 41.5 kbar.

High-pressure data for TeDi were collected at 293(2) K on a Rigaku XtaLAB Synergy-S diffractometer using mirror monochromated Mo Kα radiation (λ = 0.71073 Å) generated using a microfocus sealed X-ray tube source and detected at a HyPix Arc-100 Detector. A FII needle crystal was cut to dimensions of approximately 0.07 x 0.12 x 0.14 mm and studied in Daphne-7575 at pressures of 0.0, 1.2(5), 3.5(5), 13.7(5), 19.0(9), 26.3(5), 30.4(5), 34.7(5) and 41.5(5) kbar. The hydrostatic limit of Daphne-7575 is reported to be approximately 40 kbar.[11, 12]

The sample chamber of the two screw Merrill-Bassett diamond anvil cell (DAC) was formed by two 800 μm culet faces of Boehler-Almax diamonds and a stamped steel sheet (thickness 250 μm) indented to a thickness of approximately 110 μm with a gasket hole of diameter 380 μm, drilled using a BETSA electric discharge machine. In each case, the sample crystal was fixed to one culet face by means of high vacuum hydrocarbon grease alongside two ruby spheres which allowed for pressure measurement using the ruby fluorescence method.[13] After each pressure ramp, the pressure inside the DAC was allowed to equilibrate for a minimum of 6 hours before data collection was initiated. Pressure measurements were taken immediately before and after each collection, and the pressure was reported as the average.

Cell refinement and data reduction were carried out using the software CrysAlisPRO.[8] Special settings (DAC opening angle, dataset resolution limits, profile rejection parameters and regular background updates) were implemented in the data reduction step, which helped remove contaminating diamond reflections and powder rings from the data. Individual specifications of the exact settings used are contained within the individual CIFs. Multi-scan absorption corrections were applied with an empirical absorption correction using spherical harmonics, implemented in the SCALE3 ABSPACK scaling algorithm[14] through CrysAlisPRO.[8] All structures were solved by SHELXT[3] and matched the ambient pressure structure obtained on the same crystal outside of the DAC at 293 K. Structure solution and refinement were carried out using SHELXL[4] with the Olex2 interface.[2] For all high-pressure datasets, in order to preserve the data-to-parameter ratio as much as possible, all non-hydrogen atoms were refined using isotropic displacement parameters and all hydrogen atoms were generated in calculated positions and refined using a riding model. A global rigid body restraint was also applied to all high-pressure datasets.

Solution Crystallisation and TeDi Crystal Details

Terephthalic dihydrazide, TeDi (CAS 136-64-1), is a white crystalline powder supplied by Tokyo Chemical Industry (TCI) UK and was used without any further purification. Analysis calc. for $C_8H_{10}N_4O_2$: C 49.48, H 5.19, N 28.85 %, found: C 49.42, H 5.17, N 29.21 %. Water (1.0 mL) was added to TeDi powder (0.005 g, 0.026 mmol), heated with a heat gun until the solvent boiled and allowed to cool to room temperature, which yielded a concomitant solution of crystals with plate, block, accordion, and needle morphologies within minutes. The concomitant crystallization of TeDi in solution was observed at all concentrations from 1.0 to 5.0 mg/ml in water, with heating to 100 °C required to dissolve TeDi powder in each case. TeDi powder is insoluble in solvents other than water and DMSO, and only dissolves in water after heating to 100 °C.

FI Plate Crystals: FI plate crystals were extremely metastable in water; therefore, a mixture of water and ethanol (0.5:0.5 mL) was used to crystallize the small plates employing the same method mentioned above. The addition of ethanol appeared to slow the rapid twinning of the plate crystals. The FI non-twinned plate crystals were analyzed at the Diamond Light Source on instrument I-19: M = 194.20 g/mol, 0.039 × 0.034 × 0.007 mm^3, monoclinic, space group $P2_1/c$ (no. 14), a = 7.94290(10) Å, b = 13.1620(2) Å, c = 8.2047(2) Å, β = 104.1790(10) °, V

= 831.62(3) Å^3, Z = 4, D_c = 1.551 g/cm^3, μ = 0.109 mm^{-1}, F(000) = 408.0, Synchrotron radiation, λ = 0.6889 Å, T = 100.0 K, $2\theta_{max}$ = 64.218 °, 14689 reflections collected. Final GooF = 1.081, R_1 = 0.0378 (2884 reflections with I > = 2σ(I)), wR_2 = 0.1188 (all data), 151 parameters, 0 restraints. CCDC deposition number: 2494050.

FI Block Crystals: M = 194.20 g/mol, 0.097 × 0.077 × 0.052 mm^3, monoclinic, space group $P2_1/c$ (no. 14), a = 7.9643(10) Å, b = 13.1468(12) Å, c = 8.1818(10) Å, β = 104.275(12) °, V = 830.22(17) Å^3, Z = 4, D_c = 1.554 g/cm^3, μ = 0.116 mm^{-1}, F(000) = 408.0, Mo Kα radiation, λ = 0.71073 Å, T = 120.0 K, $2\theta_{max}$ = 64.982 °, 5875 reflections collected. Final GooF = 0.835, R_1 = 0.0480 (5875 reflections with I > = 2σ(I)), wR_2 = 0.1089 (all data), 152 parameters, 0 restraints. Twin law: (0 0 1, 0 -1 0, 1 0 0). Twin component scales: 0.4933(13):0.5067(13). CCDC deposition number: 2494045.

FI Accordion Crystals: M = 194.20 g/mol, 0.241 × 0.059 × 0.034 mm^3, monoclinic, space group $P2_1/c$ (no. 14), a = 8.0647(15) Å, b = 13.169(2) Å, c = 8.1042(15) Å, β = 104.289(6) °, V = 834.1(3) Å^3, Z = 4, D_c = 1.547 g/cm^3, μ = 0.116 mm^{-1}, F(000) = 408.0, Mo Kα radiation, λ = 0.71073 Å, T = 120.0 K, $2\theta_{max}$ = 50.698 °, 16278 reflections collected. Final GooF = 1.095, R_1 = 0.0656 (1562 reflections with I > = 2σ(I)), wR_2 = 0.1812 (all data), 132 parameters, 0 restraints. Twin law: (0 0 1, 0 -1 0, 1 0 0). Twin component scales: 0.547(5):0.453(5). CCDC deposition number: 2494051.

FII Needle Data: M = 194.20 g/mol, 0.498 × 0.044 × 0.035 mm^3, monoclinic, space group $P2_1/n$ (no. 14), a = 6.1936(16) Å, b = 3.7771(10) Å, c = 18.025(5) Å, β = 94.485(9) °, V = 420.39(19) Å^3, Z = 2, D_c = 1.534 g/cm^3, μ = 0.115 mm^{-1}, F(000) = 204.0, Mo Kα radiation, λ = 0.71073 Å, T = 120.0 K, $2\theta_{max}$ = 49.992 °, 7313 reflections collected). Final GooF = 1.257, R_1 = 0.0659 (750 reflections with I > = 2σ(I)), wR_2 = 0.1220 (all data), 76 parameters, 0 restraints. CCDC deposition number: 2494046.

The crystal packing in FI involves strong intermolecular NH···O hydrogen bonds to give lamellar sheets (N···O distances of 2.85 and 2.90 Å). Weaker NH···O hydrogen bonds exist between layers to give $R_2^2(10)$ dimers (N···O distances of 3.11 Å) and $C(10)$ chains (N···O distances of 3.14 Å) and weak aromatic stacking at centroid distances of 5.0 Å. FII, however, packs in a herringbone pattern with diverse hydrogen bonding. There are twelve strong intermolecular hydrogen bonds per TeDi molecule, joining eight different TeDi molecules via $R_2^2(10)$ NH···O dimers and $C(10)$ NH···N chains. FII exhibits strong aromatic stacking that dominates throughout the structure, with centroid···centroid distances of 3.78 Å that correspond to the length of the b-axis and the direction in which the TeDi molecules stack.

Gel Crystallisation and Gelator Crystal Details

The gel phase experiments were performed in a 1.8 mL vial and ranged from 1.0 to 5.0 mg/mL of TeDi in water with 4.0 mg of DBS-CONHNH$_2$ to give 0.4% wt/vol gels. The solution was heated with a heat gun until it became transparent. The solution was left to cool to ambient temperature, with the gel and crystal formation occurring overnight. Gel formation was confirmed by vial inversion in all cases.

DBS-CONHNH$_2$: The gelator was synthesized as reported in the supplementary material of reference 15. White powder, calculated to contain 1.5 water molecules: $C_{22}H_{26}N_4O_8.1.5H_2O$: C 52.69, H 5.83, N 11.17%, found: C 52.66, H 5.55, N 10.88 %. Crystal Data: M = 483.477 g/mol, monoclinic, space group $P2_1$ (no. 4), a = 4.6808(10) Å, b = 27.412(10) Å, c = 17.623(4) Å, β = 92.50(3)°, V = 2259.0(10) Å^3, Z = 4, D_c = 1.422 g/cm^3, F(000) = 380.6, electron radiation, λ = 0.0251 Å, T = 175(5) K, $2\theta_{max}$ = 1.6°, 18601 reflections collected). Final GooF = 1.351, R_1 = 19.96 (4166 reflections with I > = 2σ(I)), wR_2 = 45.88 (all data), 645 parameters, 718 restraints. CCDC deposition number: 2465455.

The structure has one resolvable water molecule per two LMWG molecules, with the hydroxyl group of the sugar unit hydrogen bonding to the water molecule, at an O···O distance of 2.77 Å. Within the asymmetric unit, the two gelator molecules are joined by a NH···O hydrogen bond between the acylhydrazide units with a N···O distance of 2.97 Å, greater than the predicted hydrogen bond distance reported by Knani and Alperstein.[16] Additional intermolecular NH···O, OH···N, and OH···O hydrogen bonds run throughout the structure as a result of the hydrogen bond donors and acceptors on the sugar units and the acylhydrazide portions of the LMWG. Additional hydrogen bond information and estimated standard deviations for DBS-CONHNH$_2$ can be found in **Table S10**. The most favourable interaction in the LMWG structure is the aliphatic stacking of the sugar groups, with a calculated energy of –126.3 kJ/mol,[17, 18] accompanied by aromatic stacking (centroid···centroid distances of 4.69 Å, the same as DBS itself (refcode DURJUY),[19] but slightly shorter than computed for DBS-CONHNH$_2$ at centroid distances of 4.73 Å).[16] The calculated PXRD diffractogram from the electron diffraction structure, related to the experimental DBS-CONHNH$_2$ powder and air-dried gel patterns, is highly comparable and can be found in **Figure S13**.

3. Computational methods

Conformer Calculations and Analysis

MP2: Each interacting pair of molecules from the crystal structure of a compound with the hydrogens normalized, was generated from Mercury[20] as a mol2 file and converted to a Gaussian 16 input file in GaussView.[21] The difference between the interacting pair total electronic energy and the sum of the total electronic energies of the two individual molecules was determined as the intermolecular interaction energy of the specific interacting pair (**Table S4**). The electronic energies were computed at MP2/6-311G(d,p) with the IEF-PCM solvation model[22] using water as solvent. Each possible TeDi conformer as starting geometry was optimized at MP2/6-311G(d,p) with the Gaussian16 package[23] to locate true minima as established by no imaginary frequencies from frequency calculations at MP2/6-311G(d,p). Twelve true minima were located as listed in **Table S2**.

DFT: Each possible TeDi conformer as starting geometry was optimized at PBE0/Def2TZVPP with the Gaussian 16[23] to locate true minima as established by no imaginary frequencies from frequency calculations at PBE0/Def2TZVPP applying the IEF-PCM model[22] using water as solvent and the Grimme dispersion correction, GD3BJ.[24] Twelve true minima were located (**Table S3**). Each interacting pair of molecules from the crystal structure of a compound, with

the hydrogens normalized, was generated from Mercury[20] as a mol2 file and converted to a Gaussian 16 input file in GaussView.[21] The difference between the interacting pair total electronic energy and the sum of the total electronic energies of the two individual molecules was determined as the intermolecular interaction energy of the specific interacting pair (**Table S5**).

Lattice Energy Calculations

Computed lattice energy calculations involved molecular and pseudopotential plane-wave density functional theory (DFT) to obtain the inter- and intramolecular energies. Intermolecular energies are computed from a five-stage periodic crystal-phase geometry optimization with the Vienna *ab initio* Simulation Package (VASP 6.3.2) code[25] with the exchange-correlation modelled using the GGA functional Perdew-Burke-Ernzerhof (PBE).[26] The first four stages included the structural relaxation of atomic positions and unit cells to obtain good energetics and geometries, achieved by using Grimme's D2[27] (PBE-D2) and Tkatchenko-Scheffler (PBE-TS) dispersion corrections,[28] both of which account for long-range interactions such as van der Waals forces in the bulk crystal. A further single-point energy calculation was carried out with the more expensive Many-Body Dispersion (MBD) correction.[29, 30] All periodic simulations were modelled using a plane wave cut-off of 520 eV and Γ-centred Monkhorst-Pack k-point mesh[31] with a spacing of 0.03 $Å^{-1}$ sampling the first Brillouin zone.

Intramolecular energies for both forms were computed in the gas phase with periodic and molecular DFT methods in VASP and Gaussian 16, respectively.[23, 32] Where periodic DFT was used, a large simulation cell of dimensions 30×30×30 Å was constructed to reproduce an isolated molecule. The large unit cell dimensions break periodicity, preventing nearest-neighbor interactions. A multistep minimization using the exchange-correlation (XC) functional PBE in combination with the TS dispersion correction was done. The resulting optimized conformer structure was pipelined into a SPE with PBE-MBD in VASP and a SPE with double hybrid XC functional and basis set B2PLYPD/Def2TZVPP[33] in Gaussian 16.[23] Lattice energies predicted from the hybrid inter/intramolecular energy models are found in **Table S6-Table S8**. The intermolecular energy model PBE-TS-MBD remains constant with varying intramolecular energy models. Intramolecular energies are calculated as a summation of contributions from conformational adjustment and change interactions from the conformers in the gas phase.[34]

Crystal Structure Prediction

CSP Candidate Generation: The global search stage was performed with CrystalPredictorII (version 2.4.4)[35-39] to identify theoretical crystal structures as a minima in the lattice energy landscape. Four dihedral angles were treated as flexible – two at each side of the molecule: those centered on N–N bonds and those centered on carbonyl and aromatic C–C bonds. Electrostatic interactions were modelled from point charges fitted to the isolated-molecule charge density via the ChelpG method[40] computed at the PBE0/6-311+G(d,p) level of theory using Gaussian 16.[23] Repulsion–dispersion interactions were quantified via the FIT potential,[41]

with all CrystalPredictor input parameters left at their default values. A total of half a million Z'=1 initial crystals were generated using a quasi-random Sobol' sequence.[42] Minimized crystal structures within 20 kJ/mol of the global minimum were clustered using the CSD Python API,[43] resulting in 599 unique structures that constitute the Candidate Generation landscape (**Figure S11**).

The root mean square deviation of atomic positions between two 20-molecule shells ($RMSD_{20}$) was used to quantify similarity between predicted and experimental forms. The experimental polymorph of FII was found to match the global minimum of the Candidate Generation landscape, with an $RMSD_{20}$ of 0.545 Å. Similarly, the structure ranked 176th in energy matched the experimental FI, with an $RMSD_{20}$ of 0.484 Å. The energy difference between FI and FII is 11.373 kJ/mol, which falls within the 20.0 kJ/mol energy window for structures advanced for refinement. A summary of the energetics, density and structural comparisons between predicted and experimental forms is provided in **Table S9**. Our findings demonstrate that the limited consideration of molecular flexibility and the utilized energy model were sufficient to computationally generate the two experimentally observed polymorphs of TeDi.

CSP Refinement: The structures from the Candidate Generation stage were locally minimized employing Crystal Structure Optimizer-Rigid Molecules/Flexible Molecules (CSO-RM/FM) algorithm.[44] In CSO-RM/FM, electrostatics are modelled using distributed multipole moments up to hexadecapoles, derived from the isolated-molecule electron density[45] calculated at the PBE0/6-311+G(d,p) level of theory. To better account for the hydrogen-bonded interactions within the crystalline environment, a polarizable continuum model (PCM) with a dielectric constant of $\epsilon = 3$ was employed in the Refinement stage. Repulsion–dispersion interactions were calculated using parameters specifically derived at the above-mentioned level of theory (PBE0/6-311+G(d,p) with PCM).[44] A real-space cutoff of 10 Å was applied to electrostatic interactions, while a 15 Å cutoff was used for the repulsion–dispersion interactions. Additionally, extended treatment of molecular flexibility was introduced. Ten torsional angles involving at least two atoms outside the benzoic ring were allowed to vary during the lattice energy minimization.

Of the 599 structures subjected to local minimization, 10 failed to converge and were therefore excluded from further analysis. The remaining 589 minimized structures were clustered with CSD python API, resulting in 311 unique crystals depicted in **Figure S11**. Structures ranked 5th and 96th in the Refinement landscape match the experimental FI and FII, using an angle tolerance of 30 ° and a distance tolerance of 30 %, with an $RMSD_{20}$ of 0.440 Å and 0.141 Å, respectively. These lower $RMSD_{20}$ values, compared to those obtained in the candidate-generation stage, indicate that the CSO-RM/FM algorithm reproduces experimental crystal geometries with higher accuracy.

The predicted structure corresponding to FII has an energy of −149.587 kJ/mol, placing it 3.179 kJ/mol above the global minimum. The lattice energy difference between FI and FII is 11.338 kJ/mol, suggesting that FII is the thermodynamically most stable form at low temperatures in agreement with lattice energies and free energies obtained using DFT. A summary of the structural matches and lattice energies is provided in **Table S9**. Although FII is ranked 5th in the Refinement landscape compared to its position as the global minimum in the Candidate

Generation landscape, the CSP study successfully identified both experimentally observed FI and FII polymorphs, while significantly narrowing the set of plausible TeDi crystal forms.

4. References

1. F. Allen and I. Bruno, Bond lengths in organic and metal-organic compounds revisited: X-H bond lengths from neutron diffraction data, *Acta Crystallogr., Sect. B:Struct. Sci., Cryst. Eng. Mater.*, 2010, **66**, 380-386.
2. O. V. Dolomanov, L. J. Bourhis, R. J. Gildea, J. A. K. Howard and H. Puschmann, OLEX2: a complete structure solution, refinement and analysis program, *J. Appl. Crystallogr.*, 2009, **42**, 339-341.
3. G. Sheldrick, SHELXT - Integrated space-group and crystal-structure determination, *Acta Cryst. A*, 2015, **71**, 3-8.
4. G. M. Sheldrick, Crystal structure refinement with SHELXL, *Acta Cryst. C*, 2015, **71**, 3-8.
5. L. Bourhis, O. Dolomanov, R. Gildea, J. Howard and H. Puschmann, The anatomy of a comprehensive constrained, restrained refinement program for the modern computing environment-Olex2 dissected, *Acta Crystallogr., Sect. A:Found. Adv.*, 2015, **71**, 59-75.
6. G. Winter, xia2: an expert system for macromolecular crystallography data reduction, *J. Appl. Cryst.*, 2010, **43**, 186-190.
7. G. Winter, D. Waterman, J. Parkhurst, A. Brewster, R. Gildea, M. Gerstel, L. Fuentes-Montero, M. Vollmar, T. Michels-Clark, I. Young, N. Sauter and G. Evans, DIALS: implementation and evaluation of a new integration package, *Acta Crystallogr., D*, 2018, **74**, 85-97.
8. CrysAlisPro, Rigaku Oxford Diffraction/Agilent Technologies UK Ltd., 2024.
9. G. Sheldrick, A short history of SHELX, *Acta Crystallogr., Sect. A:Found. Adv.*, 2008, **64**, 112-122.
10. A. Saha, S. Nia and J. Rodríguez, Electron Diffraction of 3D Molecular Crystals, *Chem. Rev.*, 2022, **122**, 13883-13914.
11. K. Murata and S. Aoki, Development of high-pressure liquid media with good hydrostatic pressure, *High Press. Sci. Technol.*, 2016, **26**, 3-7.
12. D. Stasko, J. Prchal, M. Klicpera, S. Aoki and K. Murata, Pressure media for high pressure experiments, Daphne Oil 7000 series, *High Press. Res.*, 2020, **40**, 525-536.
13. J. D. Barnett, S. Block and G. J. Piermarini, An Optical Fluorescence System for Quantitative Pressure Measurement in the Diamond-Anvil Cell, *Rev. Sci. Instrum.*, 1973, **44**, 1-9.
14. R. Clark and J. Reid, The analytical calculation of absorption in multifaceted crystals, *Acta Cryst.*, 1995, **A51**, 887-897.
15. B. O. Okesola and D. K. Smith, Versatile supramolecular pH-tolerant hydrogels which demonstrate pH-dependent selective adsorption of dyes from aqueous solution, *Chem. Commun.*, 2013, **49**, 11164-11166.
16. D. Knani and D. Alperstein, Simulation of DBS, DBS-COOH, and DBS-CONHNH$_2$ as Hydrogelators, *J. Phys. Chem. A*, 2017, **121**, 1113-1120.
17. A. Gavezzotti, Are Crystal Structures Predictable?, *Acc. Chem. Res.*, 1994, **27**, 309-314.
18. A. Gavezzotti and G. Filippini, Geometry of the intermolecular X-H...Y (X, Y=N, O) hydrogen-bond and the calibration of empirical hydrogen-bond potentials, *J. Phys. Chem.*, 1994, **98**, 4831-4837.

19. V. M. Sánchez-Pedregal, B. Dacuña, F. Berride, E. Cabrita, A. Navarro-Vázquez, M. Kertesz, R. D. Weiss and M. Magdalena Cid, CCDC 1998331: Experimental Crystal Structure Determination, *CSD Communication*, 2020.
20. C. Macrae, I. Sovago, S. Cottrell, P. Galek, P. McCabe, E. Pidcock, M. Platings, G. Shields, J. Stevens, M. Towler and P. Wood, Mercury 4.0: from visualization to analysis, design and prediction, *J. Appl. Crystallogr.*, 2020, **53**, 226-235.
21. R. Dennington, T. Keith and J. Millam, GaussView, Version 6, 2019.
22. J. Tomasi, B. Mennucci and E. Cancès, The IEF version of the PCM solvation method: an overview of a new method addressed to study molecular solutes at the QM ab initio level, *J. Mol. Struct.:THEOCHEM*, 1999, **464**, 211-226.
23. M. J. Frisch, G. W. Trucks, H. B. Schlegel, G. E. Scuseria, M. A. Robb, J. R. Cheeseman, G. Scalmani, V. Barone, G. A. Petersson, H. Nakatsuji, X. Li, M. Caricato, A. V. Marenich, J. Bloino, B. G. Janesko, R. Gomperts, B. Mennucci, H. P. Hratchian, J. V. Ortiz, ... and D. J. Fox, Gaussian 16 Rev. C.01*Journal*, 2016.
24. S. Grimme, S. Ehrlich and L. Goerigk, Effect of the Damping Function in Dispersion Corrected Density Functional Theory, *J. Comput. Chem.*, 2011, **32**, 1456-1465.
25. G. Kresse and J. Hafner, Ab Initio Molecular-Dynamics for Liquid-Metals, *Phys. Rev. B*, 1993, **47**, 558-561.
26. J. Perdew, K. Burke and M. Ernzerhof, Generalized gradient approximation made simple, *Phys. Rev. Lett.*, 1996, **77**, 3865-3868.
27. S. Grimme, Semiempirical GGA-type density functional constructed with a long-range dispersion correction, *J. Comput. Chem.*, 2006, **27**, 1787-1799.
28. A. Tkatchenko and M. Scheffler, Accurate Molecular Van Der Waals Interactions from Ground-State Electron Density and Free-Atom Reference Data, *Phys. Rev. Lett.*, 2009, **102**.
29. A. Tkatchenko, R. DiStasio, R. Car and M. Scheffler, Accurate and Efficient Method for Many-Body van der Waals Interactions, *Phys. Rev. Lett.*, 2012, **108**.
30. A. Ambrosetti, A. Reilly, R. DiStasio and A. Tkatchenko, Long-range correlation energy calculated from coupled atomic response functions, *J. Chem. Phys.*, 2014, **140**.
31. H. Monkhorst and J. Pack, Special Points for Brillouin-Zone Integrations, *Phys. Rev. B*, 1976, **13**, 5188-5192.
32. T. Schwabe and S. Grimme, Double-hybrid density functionals with long-range dispersion corrections: higher accuracy and extended applicability, *Phys. Chem. Chem. Phys.*, 2007, **9**, 3397-3406.
33. F. Weigend, Accurate Coulomb-fitting basis sets for H to Rn, *Phys. Chem. Chem. Phys.*, 2006, **8**, 1057-1065.
34. A. Cruz-Cabeza and J. Bernstein, Conformational Polymorphism, *Chem. Rev.*, 2014, **114**, 2170-2191.
35. P. Karamertzanis, Prediction of crystal structure of molecular solids. PhD Thesis, Imperial College London (University of London), 2004.
36. P. Karamertzanis and C. Pantelides, Ab initio crystal structure prediction-I: Rigid molecules, *J. Comput. Chem.*, 2005, **26**, 304-324.
37. M. Habgood, I. Sugden, A. Kazantsev, C. Adjiman and C. Pantelides, Efficient Handling of Molecular Flexibility in Ab Initio Generation of Crystal Structures, *J. Chem. Theory Comput.*, 2015, **11**, 1957-1969.
38. I. Sugden, C. Adjiman and C. Pantelides, Accurate and efficient representation of intramolecular energy in ab initio generation of crystal structures. I. Adaptive local approximate models, *Acta Crystallogr., Sect. B:Struct. Sci., Cryst. Eng. Mater.*, 2016, **72**, 864-874.

39. I. Sugden, C. Adjiman and C. Pantelides, Accurate and efficient representation of intramolecular energy in ab initio generation of crystal structures. II. Smoothed intramolecular potentials, *Acta Crystallogr., Sect. B:Struct. Sci., Cryst. Eng. Mater.*, 2019, **75**, 423-433.
40. C. Breneman and K. Wiberg, Determining atom-centered monopoles from molecular electrostatic potentials - the need for high sampling density in formamide conformational analysis, *J. Comput. Chem.*, 1990, **11**, 361-373.
41. D. Williams, Improved intermolecular force field for molecules containing H, C, N, and O atoms, with application to nucleoside and peptide crystals, *J. Comput. Chem.*, 2001, **22**, 1154-1166.
42. I. Sobol', On the distribution of points in a cube and the approximate evaluation of integrals, *Zhurnal Vychislitel'noi Matematiki i Matematicheskoi Fiziki*, 1967, **7**, 784–802.
43. J. A. Chisholm and S. Motherwell, <i>COMPACK</i>:: a program for identifying crystal structure similarity using distances, *J. Appl. Cryst.*, 2005, **38**, 228-231.
44. D. Bowskill, B. Tan, A. Keates, I. Sugden, C. Adjiman and C. Pantelides, Large-Scale Parameter Estimation for Crystal Structure Prediction. Part 1: Dataset, Methodology, and Implementation, *J. Chem. Theory Comput.*, 2024, **20**, 10288-10315.
45. A. Stone, Distributed multipole analysis: Stability for large basis sets, *J. Chem. Theory Comput.*, 2005, **1**, 1128-1132.
